# Author Correction: Specification of neural circuit architecture shaped by context-dependent patterned LAR-RPTP microexons

**DOI:** 10.1038/s41467-024-50606-4

**Published:** 2024-07-23

**Authors:** Kyung Ah Han, Taek-Han Yoon, Jinhu Kim, Jusung Lee, Ju Yeon Lee, Gyubin Jang, Ji Won Um, Jong Kyoung Kim, Jaewon Ko

**Affiliations:** 1 Department of Brain Sciences, Daegu Gyeongbuk Institute of Science and Technology (DGIST), Daegu, 42988 Korea; 2 Center for Synapse Diversity and Specificity, DGIST, Daegu, 42988 Korea; 3 Department of New Biology, DGIST, Daegu, 42988 Korea; 4 Korea Basic Science Institute, Research Center for Bioconvergence Analysis, Cheongju, 28119 Korea; 5 Department of Life Sciences, Pohang University of Science and Technology (POSTECH), Pohang, 37673 Korea

**Keywords:** Neurotransmitters, Neural circuits, Molecular neuroscience

Correction to: *Nature Communications* 10.1038/s41467-024-45695-0, published online 22 February 2024

The original version of this Article contained errors in the sixth and seventh sentences of the abstract, which incorrectly used the term ‘objection-location memory’. The correct version states ‘object-location memory’ in place of ‘objection-location memory’. This has been corrected in both the PDF and HTML versions of the Article.

