# Supplementary material for: Specification of neural circuit architecture shaped by context-dependent patterned LAR-RPTP microexons
Source: Nat Commun. 2024 Feb 22;15:1624. doi: 10.1038/s41467-024-45695-0 (PMC10883964; doi:10.1038/s41467-024-45695-0)

Supplementary Information

Specification of neural circuit architecture shaped by context-dependent patterned LAR-RPTP microexons

Kyung Ah Han, Taek-Han Yoon *et al.*

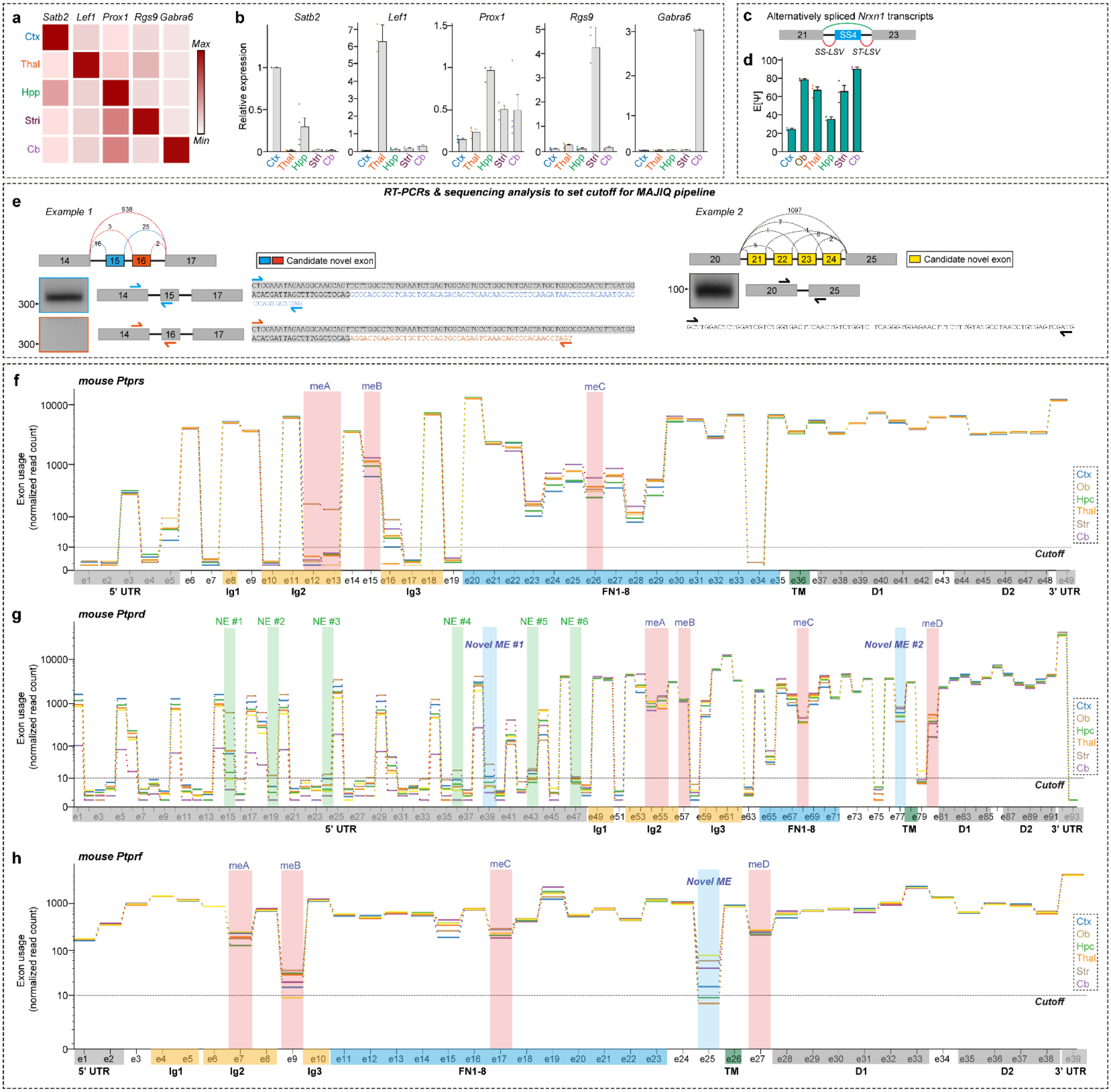

a
Satb2 Lef1 Prox1 Rgs9 Gabra6
Ctx
Thal
Hpp
Stri
Cb
Max
Min
b
Relative expression
Satb2
Lef1
Prox1
Rgs9
Gabra6
c
Alternatively spliced Nrxn1 transcripts
21
SS4
23
SS-LSV
ST-LSV
d
E[Ψ]
Ctx Ob Thal Hpp Stri Cb
RT-PCRs & sequencing analysis to set cutoff for MAJIQ pipeline
e
Example 1
Candidate novel exon
Example 2
Candidate novel exon
f
mouse Ptprs
Exon usage (normalized read count)
meA
meB
meC
Cutoff
Ctx
Ob
Hpc
Thal
Str
Cb
5' UTR
Ig1
Ig2
Ig3
FN1-8
TM
D1
D2
3' UTR
g
mouse Ptprd
NE #1
NE #2
NE #3
NE #4
NE #5
NE #6
Novel ME #1
Novel ME #2
meA
meB
meC
meD
Cutoff
h
mouse Ptprf
meA
meB
meC
Novel ME
meD
Cutoff

Supplementary Figure 1. Determination of the cutoff for MAJIQ-based alternative splicing analysis, analyses of brain region-specific *Nrxn1* splicing at SS#4, and alternative splicing landscapes of LAR-RPTP mRNAs

a, **b** Validation of each brain subregion used for RNA-seq. Summary heatmap (**a**) and summary graphs of quantitative RT-PCR analyses using tissue-specific probes (**b**). The following tissue-specific probes were used: *Satb2* (for cortex), *Lef1* (for thalamus), *Prox1* (for hippocampus), *Rgs9* (for striatum), and *Gabra6* (for cerebellum). Data are presented as means ± SEMs (n = 3 mice for all experimental groups; except for *Satb2* and *Prox1*, n = 4 mice).

c, **d** Schematic illustration (**c**) of *Nrxn1* SS#4-associated LSVs, and summary data (**d**) showing quantification of SS#4 (e22) across six brain regions. The bars indicate the standard error across triplicates for each region (n = 3 biological replicates). Abbreviations: Cb, cerebellum; Ctx, cortex; Hpp, hippocampus; Ob, olfactory bulb; Stri, striatum; Thal, thalamus.

e Confirmation of the arbitrarily determined cutoff value (10) by RT-PCR analysis. Exemplary representative gel images obtained using probes targeting the indicated flanking exons are shown. Note that only exon 15 (value 15), but not exon 16 (value 2), exon 21 (value 4), exon 22 (value 4), exon 23 (value 1), or exon 24 (value 7), was robustly amplified, consistent with MAJIQ-based bioinformatics analysis.

f–h Visualization of the alternative splicing repertoire of mouse LAR-RPTP coding genes (*Ptprs*, *Ptprd*, and *Ptprf*) across six different brain areas using *DEXSeq*. Abbreviations: me, microexon; NE, novel exon; Novel ME, novel microexon; D1 and D2, phosphatase domains; FN, fibronectin type III repeat; Ig, immunoglobulin domain; TM, transmembrane domain; UTR, untranslated region. Source data are provided as a Source Data File.

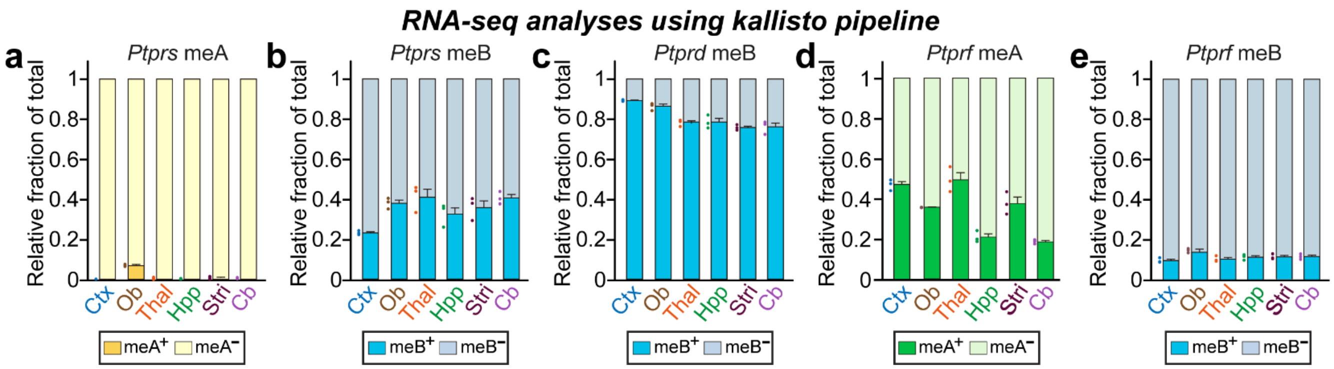

Supplementary Figure 2. Isoform abundance of LAR-RPTP mRNAs, as assessed using kallisto

Percentage of total isoforms with and without microexons across LAR-RPTP genes (*Ptprs* meA, **a**; *Ptprs* meB, **b**; *Ptprd* meB, **c**; *Ptprf* meA, **d**; *Ptprf* meB, **e**). Values are expressed as means ± SEMs (n = 3 mice for all experimental groups). Source data are provided as a Source Data File.

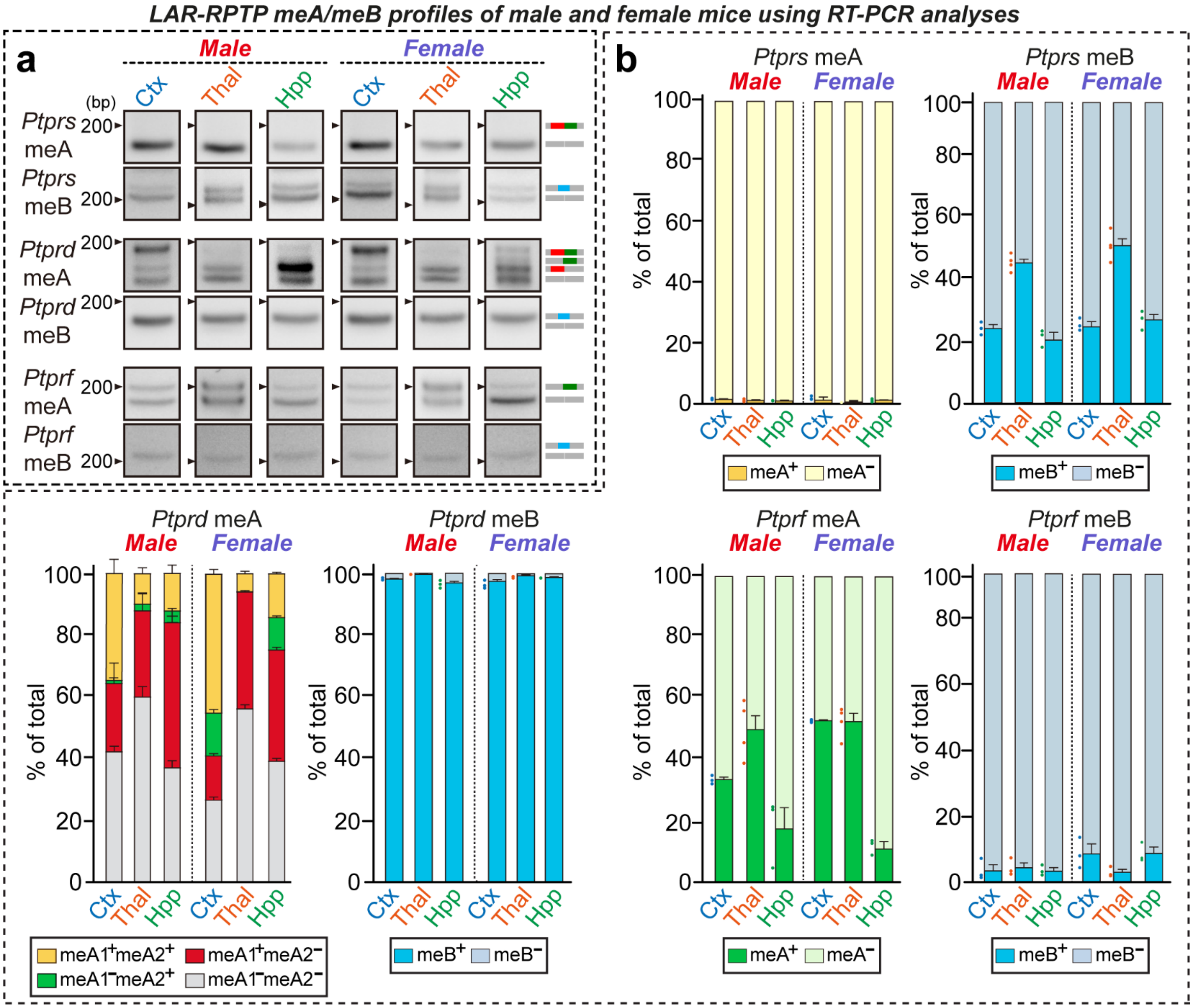

Supplementary Figure 3. Expression profiles of mouse LAR-RPTP microexons in male and female brains

a Representative DNA-PAGE gel images of LAR-RPTP microexon expression analyses in the cortex (Ctx), thalamus (Thal), and hippocampus (Hpp) of adult male and female mice.

b Summary data showing quantification of LAR-RPTP meA and meB microexons in the indicated brain areas. Data are presented as means ± SEMs (n = 3 mice for all experimental groups; except for *Ptprs* meA [male: Thal, female: Thal], *Ptprd* meA [male: Ctx and Hpp, female: Ctx and Hpp] and *Ptprf* meA [male: Thal, female: Thal], n = 4 mice). Abbreviations: Ctx, cortex; Hpp, hippocampus; Thal, thalamus. Source data are provided as a Source Data File.

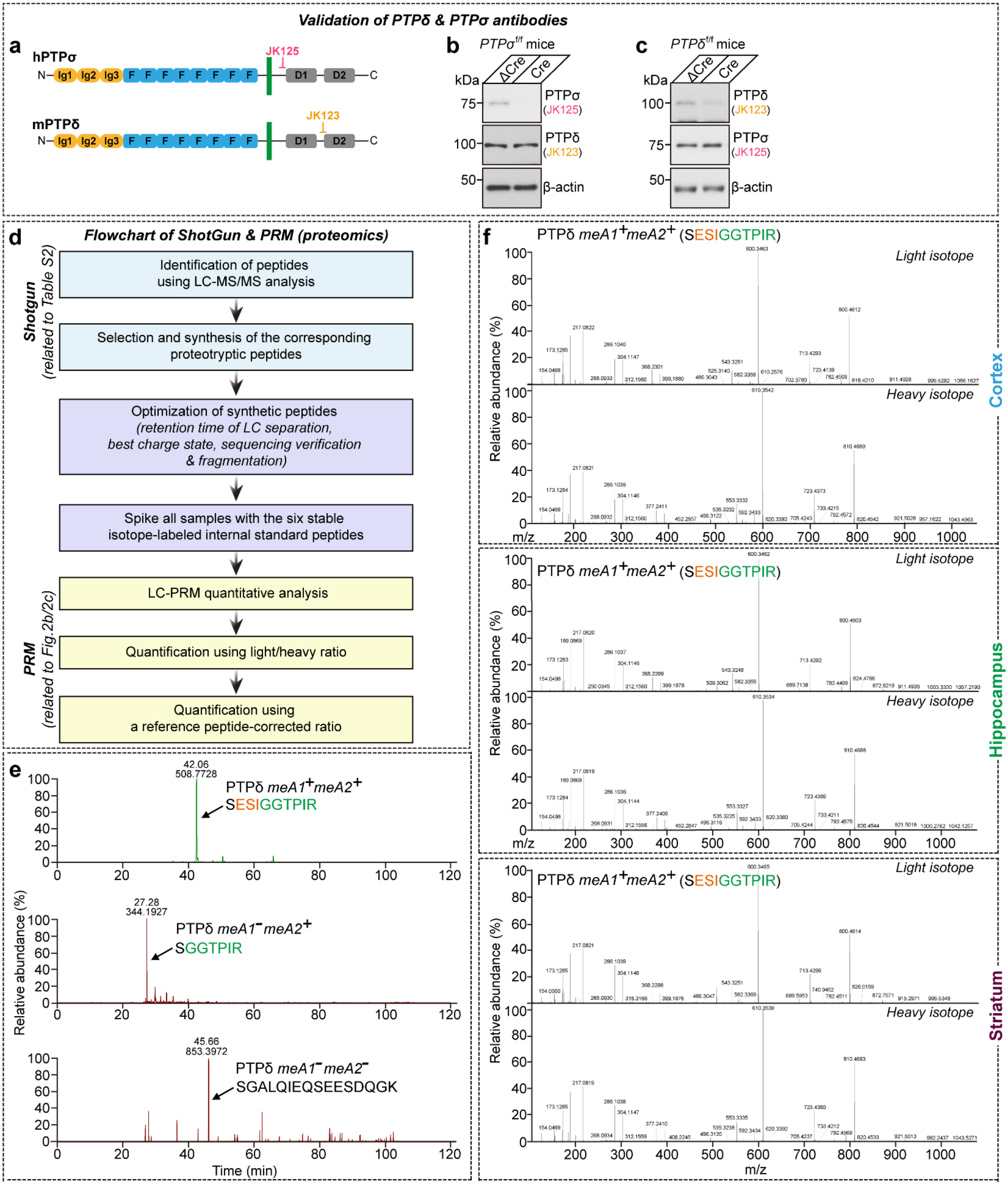

Validation of PTPδ & PTPσ antibodies
a
hPTPσ
JK125
N– Ig1 Ig2 Ig3 F F F F F F F F D1 D2 –C
mPTPδ
JK123
N– Ig1 Ig2 Ig3 F F F F F F F F D1 D2 –C
b
PTPσ f/f mice
ΔCre Cre
kDa
75
PTPσ (JK125)
100
PTPδ (JK123)
50
β-actin
c
PTPδ f/f mice
ΔCre Cre
kDa
100
PTPδ (JK123)
75
PTPσ (JK125)
50
β-actin
d
Flowchart of ShotGun & PRM (proteomics)
Shotgun (related to Table S2)
Identification of peptides using LC-MS/MS analysis
Selection and synthesis of the corresponding proteotryptic peptides
Optimization of synthetic peptides (retention time of LC separation, best charge state, sequencing verification & fragmentation)
Spike all samples with the six stable isotope-labeled internal standard peptides
PRM (related to Fig.2b/2c)
LC-PRM quantitative analysis
Quantification using light/heavy ratio
Quantification using a reference peptide-corrected ratio
e
42.06
508.7728
PTPδ meA1+meA2+
SESIGGTPIR
27.28
344.1927
PTPδ meA1−meA2+
SGGTPIR
45.66
853.3972
PTPδ meA1−meA2−
SGALQIEQSEESDQGK
Relative abundance (%)
Time (min)
f
PTPδ meA1+meA2+ (SESIGGTPIR)
Light isotope
Heavy isotope
Relative abundance (%)
m/z
Cortex
Hippocampus
Striatum

Supplementary Figure 4. Authenticity testing of anti-PTPσ and anti-PTPδ antibodies, LC-MS/MS spectra of various PTPδ-derived peptides expressed in the cortex of adult mice by Shotgun mass spectrometry analysis, and spectra of PTPδ meA1⁺ meA2⁺ peptides expressed in the cortex, hippocampus and striatum of adult mice by PRM analysis

a Schematic of epitopes of anti-PTPσ and anti-PTPδ antibodies (JK125 and JK123, respectively) used in the current study. Abbreviations: D1 and D2, phosphatase domains; FN, fibronectin type III repeat; Ig, immunoglobulin domain; N, N-terminus; C, C-terminus.

b, c Representative immunoblots of the anti-PTPσ and anti-PTPδ antibodies used in this study. Adeno-associated viruses (AAVs) expressing active Cre recombinase or inactive Cre (ΔCre) recombinase were injected into the CA1 region of adult *PTPσ* $^{f/f}$ (**b**) or *PTPδ*$^{f/f}$ (**c**) mice and the infected lysates were probed with the indicated antibodies. β-actin antibodies were used as a normalization control.

d Workflow of proteomics approaches (shotgun and PRM analyses) employed in the current study. Abbreviations: LC-MS/MS, liquid chromatography with tandem mass spectrometry; PRM, parallel reaction monitoring.

e Total ion chromatogram of tryptic digests of PTPδ–bound eluates separated by liquid chromatography (LC) and extracted ion chromatograms of m/z 508.7728 (SESIGGTPIR, 2+), 344.1927 (SGGTPIR, 2+), and 853.3972 (SGALQIEQSEESDQGK, 2+) ions generated from mouse PTPδ meA variants using shotgun analyses.

f Representative MS/MS spectra of LC-PRM for detection of peptides encoding PTPδ meA1^{+}A2^{+} from the cortex, hippocampus, and striatum of adult mice.

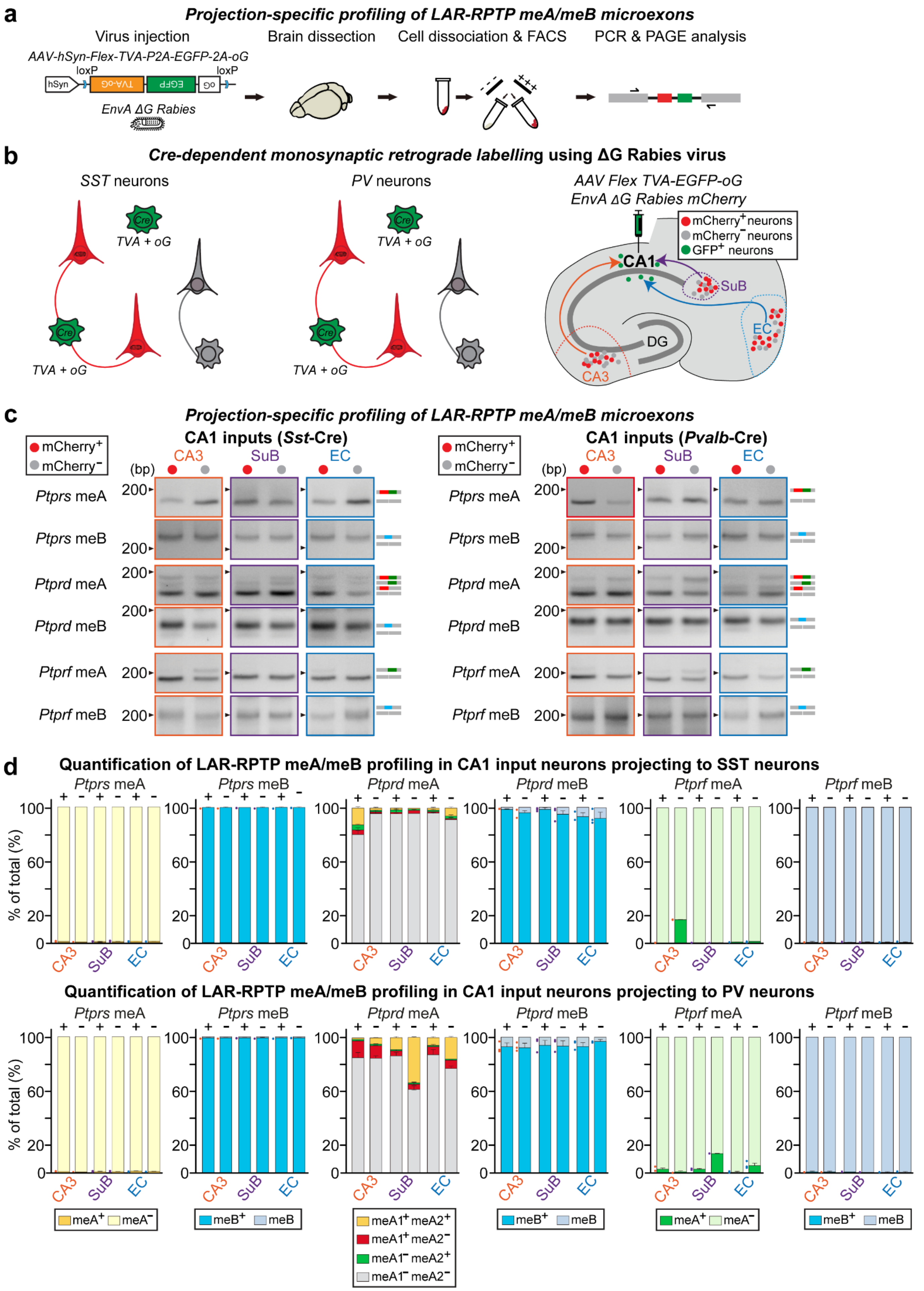
a
Projection-specific profiling of LAR-RPTP meA/meB microexons
Virus injection
Brain dissection
Cell dissociation & FACS
PCR & PAGE analysis
AAV-hSyn-Flex-TVA-P2A-EGFP-2A-oG
loxP
hSyn
TVA-oG
EGFP
oG
EnvA ΔG Rabies
b
Cre-dependent monosynaptic retrograde labelling using ΔG Rabies virus
SST neurons
PV neurons
Cre
TVA + oG
AAV Flex TVA-EGFP-oG
EnvA ΔG Rabies mCherry
mCherry+ neurons
mCherry− neurons
GFP+ neurons
CA1
SuB
EC
DG
CA3
c
Projection-specific profiling of LAR-RPTP meA/meB microexons
CA1 inputs (Sst-Cre)
CA1 inputs (Pvalb-Cre)
mCherry+
mCherry−
(bp)
CA3
SuB
EC
Ptprs meA
Ptprs meB
Ptprd meA
Ptprd meB
Ptprf meA
Ptprf meB
200
d
Quantification of LAR-RPTP meA/meB profiling in CA1 input neurons projecting to SST neurons
Quantification of LAR-RPTP meA/meB profiling in CA1 input neurons projecting to PV neurons
% of total (%)
100
60
20
0
+
−
meA+
meA−
meB+
meB
meA1+ meA2+
meA1+ meA2−
meA1− meA2+
meA1− meA2−

Supplementary Figure 5. Profiling of mouse LAR-RPTP microexon profiles in distinct hippocampal CA1 GABAergic interneurons

a Schematic showing the experimental procedure for LAR-RPTPs microexon profiling in hippocampal CA1 GABAergic (SST^+ or PV^+) interneurons.

b Cre-dependent TVA and oG expressing system for cell-type–specific retrograde labeling. TVA and oG are expressed only in Cre-expressing CA1 interneurons, and EnvA ΔG rabies is complemented in Cre-expressing cells and activated for mono-*trans*-synaptic retrograde tracing. Abbreviations: EnvA, envelop protein from avian ASLV type A; PV, parvalbumin; SST, somatostatin; TVA, EnvA receptor.

c Representative DNA-PAGE of LAR-RPTPs meA and meB microexons in CA3, subicular, and EC neurons that project to hippocampal CA1 GABAergic (SST^+ or PV^+) interneurons.

d Quantitative analyses of LAR-RPTPs meA and meB microexon expression repertoires in (**c**). Values are expressed as means ± SEMs (n = 3 mice). Abbreviations: EC, entorhinal cortex; SuB, subiculum. Source data are provided as a Source Data File.

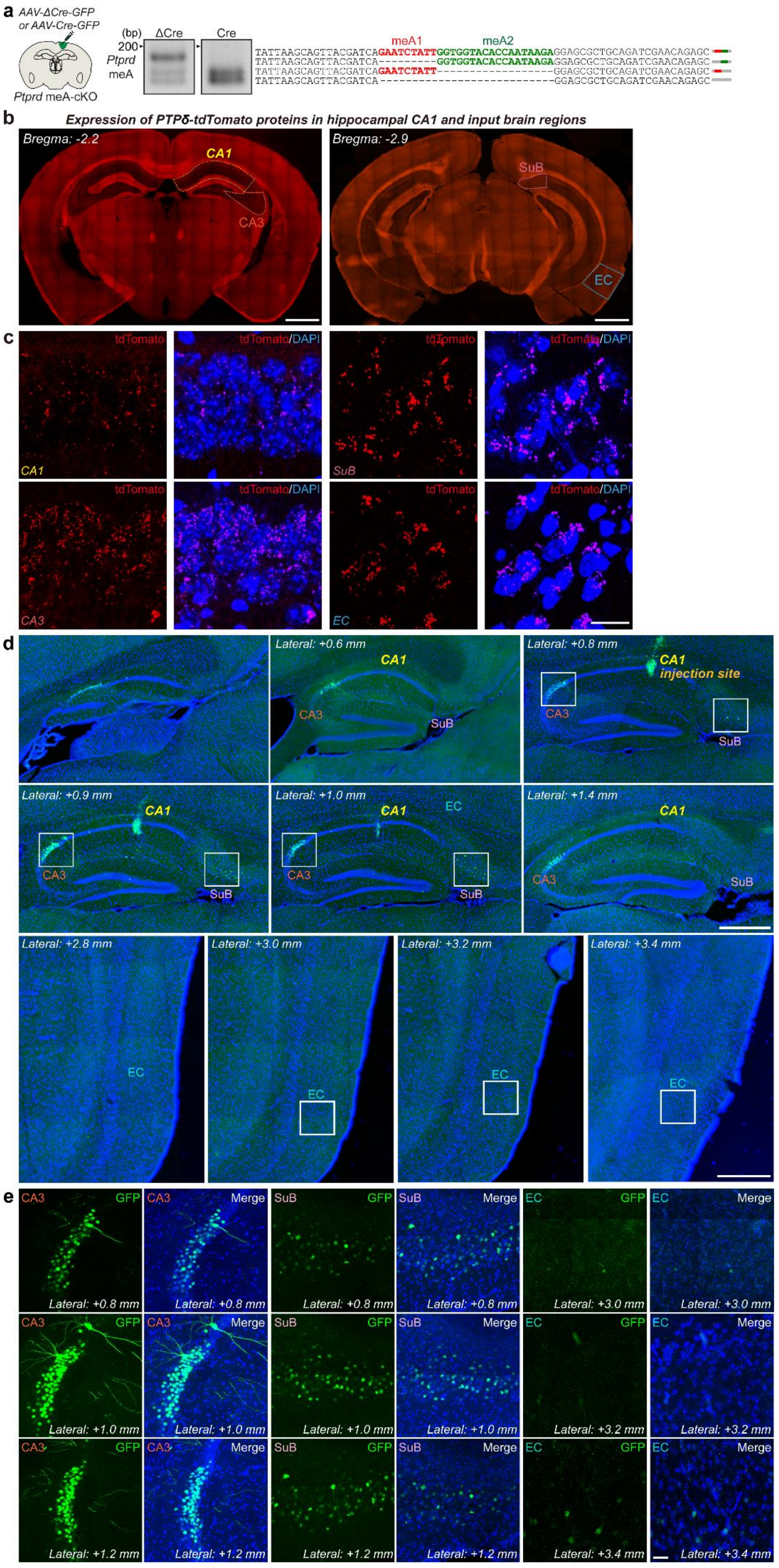
a
AAV-ΔCre-GFP
or AAV-Cre-GFP
Ptprd meA-cKO
(bp)
200
Ptprd
meA
ΔCre
Cre
meA1
meA2
TATTAAGCAGTTACGATCAGAATCTATTGGTGGTACACCAATAAGAGGAGCGCTGCAGATCGAACAGAGC
TATTAAGCAGTTACGATCA---------GGTGGTACACCAATAAGAGGAGCGCTGCAGATCGAACAGAGC
TATTAAGCAGTTACGATCAGAATCTATT------------------GGAGCGCTGCAGATCGAACAGAGC
TATTAAGCAGTTACGATCA ---------------------------GGAGCGCTGCAGATCGAACAGAGC
b
Expression of PTPδ-tdTomato proteins in hippocampal CA1 and input brain regions
Bregma: -2.2
CA1
CA3
Bregma: -2.9
SuB
EC
c
tdTomato
tdTomato/DAPI
CA1
CA3
SuB
EC
d
Lateral: +0.6 mm
Lateral: +0.8 mm
CA1
injection site
CA3
SuB
Lateral: +0.9 mm
Lateral: +1.0 mm
Lateral: +1.4 mm
EC
Lateral: +2.8 mm
Lateral: +3.0 mm
Lateral: +3.2 mm
Lateral: +3.4 mm
e
GFP
Merge
Lateral: +0.8 mm
Lateral: +1.0 mm
Lateral: +1.2 mm
Lateral: +3.0 mm
Lateral: +3.2 mm
Lateral: +3.4 mm

Supplementary Figure 6. Validation of the loss of *Ptprd* variants containing meA in cortical cells of PTPδ meA floxed mice expressing Cre recombinase, expression of PTPδ protein in three brain regions that innervate hippocampal CA1 neurons, and distribution of retrogradely labeled input neurons in the CA3, SuB or EC that project to dCA1 neurons

a Validation of *Ptprd*-meA transgenic mouse lines lacking meA conditionally, as assessed by DNA-PAGE analyses. Note that cortical cells from *Ptprd* meA-cKO mice specifically lack *Ptprd* variants containing meA.

b Low-resolution images of coronal brain sections from PTPδ-tdTomato reporter mice, showing the expression of tdTomato fluorescence across the hippocampal dCA1, CA3, subiculum (SuB), and entorhinal cortex (EC). Scale bar = 1 mm.

c Representative images of brain sections from PTPδ-tdTomato reporter mice, showing the expression of tdTomato fluorescence in the hippocampal dCA1, CA3, SuB, and EC. Scale bar = 20 μm.

d Representative images of sagittal brain sections injected with rAAV2-retro. rAAV2-retro–mediated labeling of dCA1 input neurons was detected in the CA3, SuB or EC along the sagittal axis using Axioscan. Scale bar, 500 μm. Abbreviations: EC, entorhinal cortex; GFP, green fluorescent protein; SuB, subiculum.

e Expanded views of the boxed area in panel A. Scale bar, 50 μm.

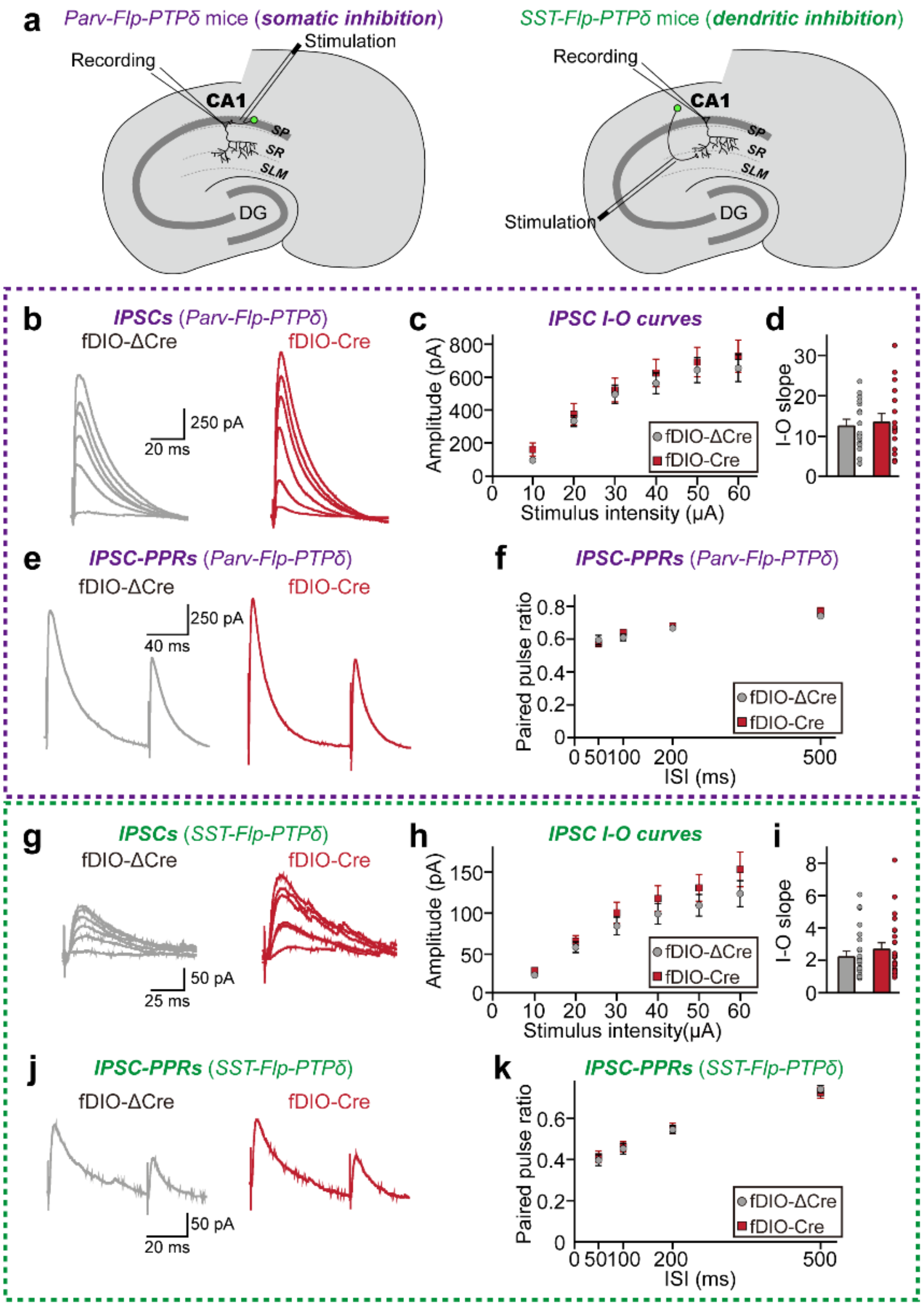

a
Parv-Flp-PTPδ mice (somatic inhibition)
SST-Flp-PTPδ mice (dendritic inhibition)
Recording
Stimulation
CA1
SP
SR
SLM
DG
b
IPSCs (Parv-Flp-PTPδ)
fDIO-ΔCre
fDIO-Cre
250 pA
20 ms
c
IPSC I-O curves
Amplitude (pA)
Stimulus intensity (μA)
fDIO-ΔCre
fDIO-Cre
d
I-O slope
e
IPSC-PPRs (Parv-Flp-PTPδ)
250 pA
40 ms
f
IPSC-PPRs (Parv-Flp-PTPδ)
Paired pulse ratio
ISI (ms)
g
IPSCs (SST-Flp-PTPδ)
50 pA
25 ms
h
IPSC I-O curves
Stimulus intensity(μA)
i
j
IPSC-PPRs (SST-Flp-PTPδ)
50 pA
20 ms
k
IPSC-PPRs (SST-Flp-PTPδ)

Supplementary Figure 7. Analysis of PTPδ function in regulating specific GABAergic neural circuits in the mouse hippocampal CA1

a Schematic of electrophysical recordings showing stimulation and recording sites in the hippocampal CA1. For recording input from PV^+ interneurons of *Parv-Flp-PTPδ* mice, the stimulation electrode was positioned on the SP layer (**left**). For recording input from SST^+ interneurons of *SST-Flp-PTPδ* mice, axon fibers on the SLM layer were electrically stimulated (**right**). Abbreviations: DG, dentate gyrus, Parv, parvalbumin; SST, somatostatin

b–d Measurement of somatic-evoked IPSCs (eIPSCs). Representative somatic eIPSC traces (**b**), average eIPSC input-output (I-O) curve (**c**), and average eIPSC I-O slope (**d**) for hippocampal CA1 pyramidal neurons from *Parv-Flp-PTPδ* mice expressing fDIO-ΔCre (black) or fDIO-Cre (red). Data are presented as means ± SEMs ('n' denotes number of cells/mice; fDIO-ΔCre, n = 17/5; fDIO-Cre, n = 18/5; two-tailed non-parametric Mann-Whitney *U* test).

e, **f** Release probability of PV^+ interneurons, measured by recording IPSC-PPRs. Representative traces of IPSC-PPRs at an interstimulus interval of 100 ms (**e**) and average of IPSC-PPRs (**f**). Data are presented as means ± SEMs ('n' denotes number of cells/mice; fDIO-ΔCre, n =17/5; fDIO-Cre, n = 18/5; two-tailed non-parametric Mann-Whitney *U* test).

g–i Measurement of dendritic eIPSCs. Representative dendritic eIPSC traces (**g**), average eIPSC I-O curve (**h**), and average eIPSC I-O slope (**i**) for hippocampal CA1 pyramidal neurons from *SST-Flp-PTPδ* mice expressing fDIO-ΔCre (black) or fDIO-Cre (red). Data are presented as means ± SEMs ('n' denotes number of cells/mice; fDIO-ΔCre, n = 27/7; fDIO-Cre, n = 26/7; two-tailed non-parametric Mann-Whitney *U* test).

j, k Release probability of SST^+ interneurons, measured by recording IPSC-PPRs. Representative traces of IPSC-PPRs at an interstimulus interval of 100 ms (**j**) and average of IPSC-PPRs (**k**). Data are presented as means ± SEMs ('n' denotes number of cells/mice; fDIO-ΔCre, n = 27/7; fDIO-Cre, n = 26/7; two-tailed non-parametric Mann-Whitney *U* test). Source data are provided as a Source Data File.

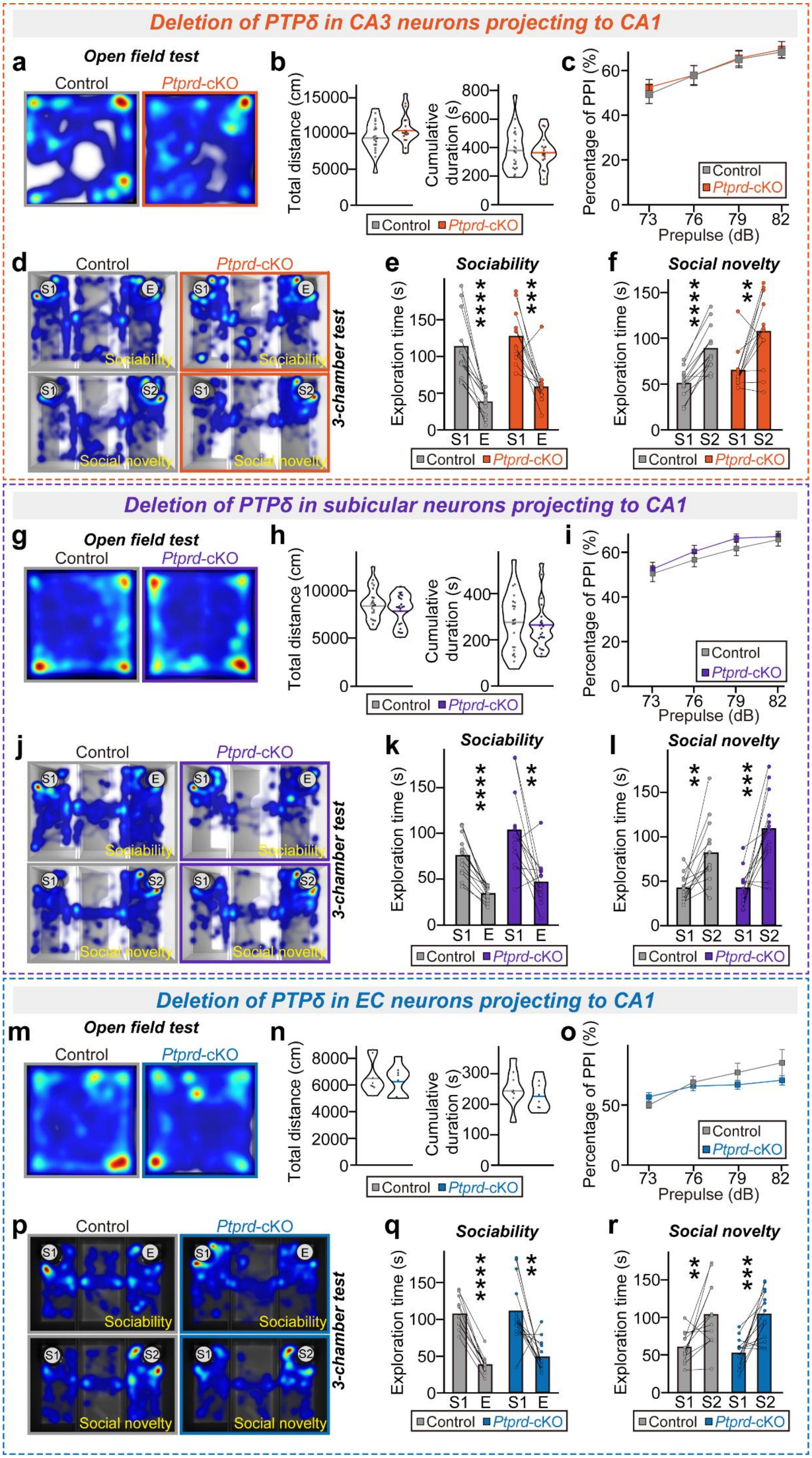

Deletion of PTPδ in CA3 neurons projecting to CA1
a
Open field test
Control
Ptprd-cKO
b
Total distance (cm)
Cumulative duration (s)
Control
Ptprd-cKO
c
Percentage of PPI (%)
Prepulse (dB)
d
3-chamber test
Sociability
Social novelty
e
Sociability
Exploration time (s)
f
Social novelty
Deletion of PTPδ in subicular neurons projecting to CA1
g
Open field test
h
i
j
k
Sociability
l
Social novelty
Deletion of PTPδ in EC neurons projecting to CA1
m
Open field test
n
o
p
q
Sociability
r
Social novelty

Supplementary Figure 8. Behavioral analyses of hippocampal circuit-specific *Ptprd*-cKO mice

a, b Analysis of locomotor activity by open-field test in CA3→dCA1 circuit-specific PTPδ-cKO mice. (a) Representative heat maps of mouse movement in the open chamber. (**b**) Analyses of total distance moved and time spent in the center zone. Data are presented as means ± SEMs ('n' denotes number of mice; Control, n = 21; *Ptprd*-cKO, n = 18; two-tailed non-parametric Mann Whitney *U*-test).

c Analysis of acoustic startle response to a sudden intense stimulus in Control and CA3→dCA1 circuit-specific PTPδ-cKO mice. Data are presented as means ± SEMs ('n' denotes number of mice; Control, n = 20; *Ptprd*-cKO, n = 18; two-way ANOVA with Sidak's multiple comparisons test).

d–f Analysis of behavior in a 3-chamber test using CA3→dCA1 circuit-specific *Ptprd*-cKO mice. Representative heat maps (**d**) of sociability and social novelty and summary graphs of sociability (**e**) and novel social recognition memory (**f**). Data are presented as means ± SEMs ('n' denotes number of mice; Control, n = 13; *Ptprd*-cKO, n = 12; $^{**}p < 0.01$, $^{***}p < 0.001$, $^{****}p < 0.0001$; two-tailed paired t test).

g, **h** Analysis of locomotor activity by open-field test in SuB→dCA1 circuit-specific *Ptprd*-cKO mice. Representative heat maps (**g**) of mouse movement in the open chamber and summary graphs (**h**). Data are presented as means ± SEMs (Control, n = 22; *Ptprd*-cKO, n = 23; two-tailed non-parametric Mann Whitney *U*-test).

i Analysis of acoustic startle response to a sudden intense stimulus in Control and SuB→dCA1 circuit-specific *Ptprd*-cKO mice. Data are presented as means ± SEMs ('n' denotes number of mice; Control, n = 22; *Ptprd*-cKO, n = 23; two-way ANOVA with Sidak's multiple comparisons test).

j–l Analysis of behavior in a 3-chamber test in SuB→dCA1 circuit-specific *Ptprd*-cKO mice. Representative heat maps (**j**) of sociability and social novelty and summary graphs of sociability (**k**) and novel social-recognition memory (**l**). Data are presented as means ± SEMs ('n' denotes number of mice; Control, n = 13; *Ptprd*-cKO, n = 13; $^{**}p < 0.01$, $^{***}p < 0.001$, $^{****}p < 0.0001$; two-tailed paired t test).

m, **n** Analysis of locomotor activity by open-field test in EC→dCA1 circuit-specific *Ptprd*-cKO mice. Representative heat maps (m) of mouse movement in the open chamber and summary graphs (**n**). Data are presented as means ± SEMs ('n' denotes number of mice; Control, n = 8; *Ptprd*-cKO, n = 8; two-tailed non-parametric Mann Whitney *U*-test).

o Analysis of acoustic startle response to a sudden intense stimulus in Control and EC→dCA1 circuit-specific *Ptprd*-cKO mice. Data are presented as means ± SEMs ('n' denotes number of mice; Control, n = 19; *Ptprd*-cKO, n = 21; two-way ANOVA with Sidak's multiple comparisons test).

p–r Analysis of behavior in a 3-chamber test in EC→dCA1 circuit-specific *Ptprd*-cKO mice. Representative heat maps (**p**) of sociability and social novelty, and summary graphs of sociability (**q**) and novel social-recognition memory (**r**). Data are presented as means ± SEMs ('n' denotes number of mice; Control, n = 12; *Ptprd*-cKO, n = 14; $^{**}p < 0.01$, $^{***}p < 0.001$, $^{****}p < 0.0001$; two-tailed paired t test). Source data are provided as a Source Data File.

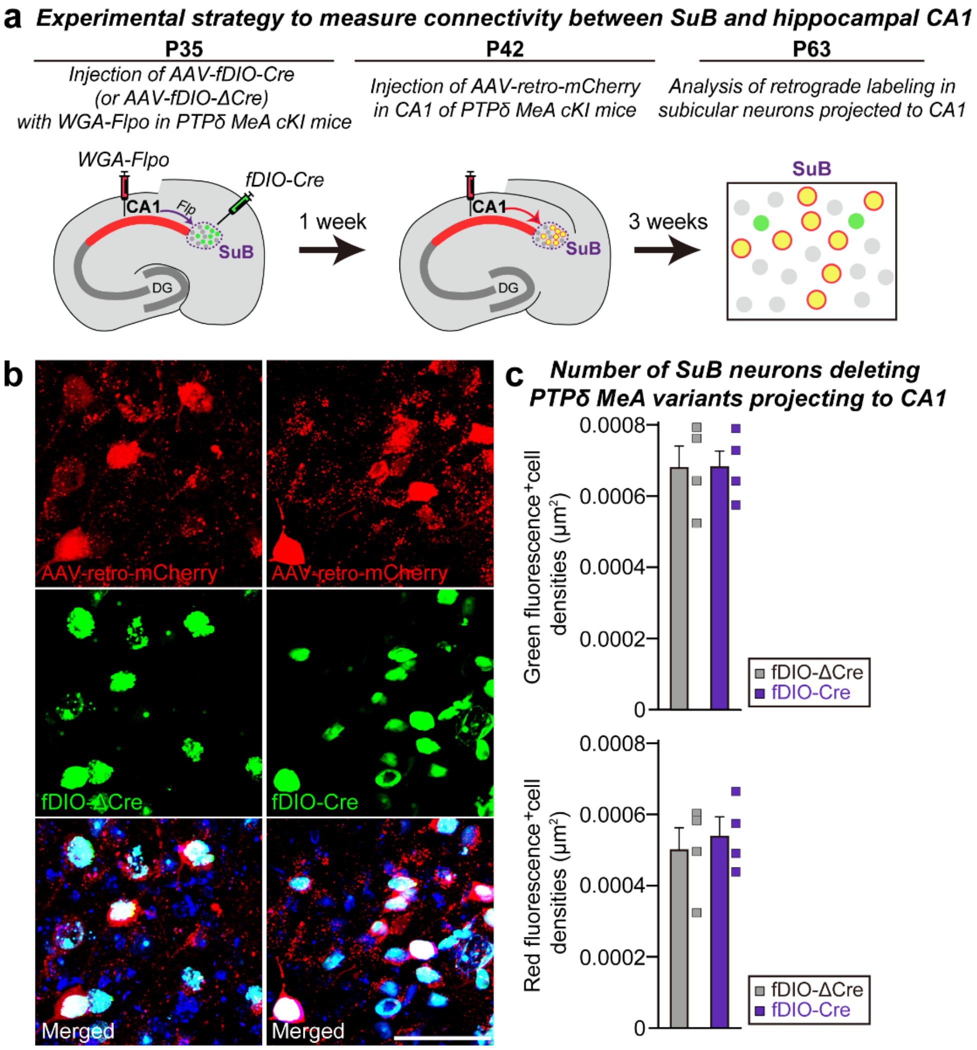

Supplementary Figure 9. Viral tracing reveals that presynaptic elimination of PTPδ meA⁺ variants in the SuB→dCA1 circuit does not affect the projection of SuB neurons onto dCA1 neurons

a Schematic showing the experimental strategy for measuring the connectivity between SuB and CA1 upon the deletion of PTPδ meA⁺ variants from SuB neurons. Abbreviations: DG, dentate gyrus; SuB, subiculum.

b Representative immunofluorescence images showing expression of fDIO-ΔCre or fDIO-Cre (green), together with expression of retrograde AAVs (red), in SuB neurons. Scale bar = 50 μm.

c Quantitative analyses of fDIO-ΔCre- or fDIO-Cre/retrograde AAV-expressing neurons, presented in (**b**). Data are presented as means ± SEMs (n = 4 mice for all experimental groups). Source data are provided as a Source Data File.

Supplementary Table 1. Prior studies investigating microexons of *neurexin* and LAR-RPTP mRNAs

RNA-seq methods	Read counts	*Nrxn* microexon detection	LAR-RPTPs microexon detection	Reference
PacBio long-read sequencing	A total of 23,943 full-length mRNA reads	O	×	1
Target-enrichment SMART sequencing	150-bp long and >10 million paired-end reads/cell	O	×	2
Patch single cell qRT profiling	N.D.	O	×	3
Single-cell RNA-seq	N.D.	N.D.	N.D.	4
In situ sequencing	N.D.	×	×	5
Ribotag immunoprecipitation sequencing	>100 million reads per biological replicate	×	×	6
Single-cell RNA-seq	10 million/cell	O	×	7
Bulk RNA-seq	N.D.	×	×	8
Single-cell RNA-seq	60,000 reads/cell; the actual median achieved was 59,728 reads/cell across 175 libraries	×	×	9
Single-cell RNA-seq	New pipeline	O	O	10

Abbreviations: N.D., not determined; RNA-seq, RNA sequencing

Supplementary Table 2. Cross-correlation score (X_{corr}) of chemically synthesized peptides used in this study

Name	Peptide	Cortex			Hippocampus		
		X_{corr}	Number of identified peptide spectral matches	Peptide sequence coverage (%)	X_{corr}	Number of identified peptide spectral matches	Peptide sequence coverage (%)
PTPδ meA1^{-}A2^{-}	SGALQIEQSE ESDQGK	2.992	1	50.0	4.6273	1	87.5
PTPδ meA1^{-} A2^{+}	SGGTPIR	1.7892	1	100.0	N.A.	N.D.	N.A.
PTPδ meA1^{+}A2^{-}	SESIGALQIEQ SEESDQGK	N.A.	N.D.	N.A.	N.A.	N.D.	N.A.
PTPδ meA1^{+}A2^{+}	SESIGGTPIR	2.366	7	90.0	2.2187	3	90.0
PTPδ exon 60	NVLELNDVR	2.3847	3	88.9	2.3675	1	88.9
PTPδ exon 61	VVAVNNIGR	2.1059	2	100.0	2.0791	2	100.0

Abbreviations: N.A., not applicable; N.D., not detected. Peptide sequence coverage (%) was calculated as [(number of matched fragment ions from LC-MS/MS analysis)/(number of amino acids in each peptide)] x 100

Supplementary Table 3. Oligonucleotide sequences for genotyping and RT-PCR experiments described in this study

**Genotyping**	**Forward**	**Reverse**
Ptprd-cKO	5′-AATTGGGACTGTGCTCCACAACTCT -3′	5′-ATGTAAGGGCCTCCTTGAAGACAGC-3′
Ptprs-cKO	5′-CACATACTCCTTCCTCTCCAAACGG-3′	5′-AGACACACTCTGAGCCCTCTGAGC-3′
Ptprd-meA cKI (WT)	5′-TGTCTTAAAAGTCAAAGAATGACTCCCC-3′	5′-ATCACTGCTCGAGGACCTCTGGATA-3′
Ptprd-meA cKI (KO)	5′-TGTCTTAAAAGTCAAAGAATGACTCCCC-3′	5′-GGCCCACACAGTAGCTGTGGCAATA-3′
Emx1-Cre	WT: 5′-AAGGTGTGGTTCCAGAATCG-3′<br>Mut: 5′-GCGGTCTGGCAGTAAAAACTATC-3′	WT: 5′-CTCTCCACCAGAAGGCTGAG-3′<br>Mut: 5′-GTGAAACAGCATTGCTGTCAC TT-3′
Pvarb-Cre	WT: 5′-CAGAGCAGGCATGGTGACTA-3′<br>Mut: 5′-GCGGTCTGGCAGTAAAAACTATC-3′	WT: 5′-AGTACCAAGCAGGCAGGAGA-3′<br>Mut: 5′-TGAAACAGCATTGCTGTCACTT-3′
Sst-Cre	WT: 5′-TCTGAAAGACTTGCGTTTGG-3′<br>Mut: 5′-TGGTTTGTCCAAACTCATCAA-3′	5′-GGGCCAGGAGTTAAGGAAGA-3′
Drd1-Cre	5′-GCTATGGAGATGCTCCTGATGGAA-3′	5′-CGGCAAACGGACAGAAGCATT-3′
Drd2-Cre	5′-GTGCGTCAGCATTTGGAGCAA-3′	5′-CGGCAAACGGACAGAAGCATT-3′
**qPCR**	**Forward**	**Reverse**
Satb2	5′-CAGAGGTACCACGTGAAGCA-3′	5′-GCCTGCGGAGTTCACATTAT-3′
Prox1	5′-TTGCAACGCTCTTTTGAATG-3′	5′-CCCCTTGTGATGAAGGAAAA-3′
Rgs9	5′-AGAAATGCTGGCCAAAGCTA-3′	5′-GCAGCTCCTTTTTGAGTTGG-3′
Lef1	5′-CGGTTGTTTCGGAAAAAGAA-3′	5′-GGTCGCTGGCTTTCTAGTTG-3′
Gabra6	5′-AGGAGTCAGTCCCAGCAAGA-3′	5′-GTTGACAGCTGCGAATTCAA-3′
Gapdh	5′-ACATGGTCTACATGTTCCAG-3′	5′-TCGCTCCTGGAAGATGGTGAT-3′
**RT-PCR**	**Forward**	**Reverse**
Ptprs meA	5'-GAGGTTGGCAGGTGATGAGT-3'	5'-TAAGGACTTCCTGCCTGTGG-3'
Ptprs meB	5'-TGAGTCCTTGACATCCGTGA-3'	5'-ACTCATCACCTGCCAACCTC-3'
Ptprd meA	5'-TGGTGGCAACACACTCGTAT-3'	5'-CGGATCCAGAAATCACTTGG-3'
Ptprd meB	5'-TACACTTCCACCTGGCATGA-3'	5'-CCGACCAAGGAAAATACGAG-3'
Ptprf meA	5'-TCGCTGCTCTCTATCTGCAA-3'	5'-CGACTATCGACATGGGACCT-3'
Ptprf meB	5'-GCCCATCATCCATTTCACAT-3'	5'-AGAGAGCAGCGAGGAGTCTG-3'
Nrxn1 SS#4	5'-CTGGCCAGTTATCGAACGCT-3'	5'-GCGATGTTGGCATCGTTCTC-3'
Ptprd e14	5'-CTCGAAATAGAAGGCAAGCA-3'	–
Ptprd e15	–	5'-CTGAGCACTGGGTGCATTT-3'
Ptprd e16	–	5'-ACTAGGTTGTGGGCTGTT-3'
Ptprd e20	–	5'-GCTTGGACTCTGGATCGT-3'
Ptprd e25	5'-CATCGACTCACAGGTTAGG-3'	–
Ptprd e60	5′-ACCCACTAATCATGAAATCA-3′	5′-CGTCGACATAGCAACA-3′
Ptprd e61	5'-CATCGACTCACAGGTTAGG-3'	5′-CTCTATACCCTTGGATCTG-3′

Supplementary Table 4. Information on peptides used for LC-PRM analyses

Peptide	Light (L) or heavy (H)	Peptide sequence	Monoisotopic mass	m/z Value for LC-PRM analysis (charge)	Fragment ions for quantification
PTPδ meA1⁻A2⁻	L	SGALQIEQSEESDQGK	1705.7875	853.3974 (2+)	y^{8}, y^{10}, y^{11}
	H	SGALQIEQSEESDQGK [Label: 13C(6)15N(2)]	1713.8017	857.4045 (2+)	
PTPδ meA1⁺A2⁺	L	SESIGGTPIR	1016.5371	508.7722 (2+)	y^{6}, y^{7}, y^{8}
	H	SESIGGTPIR [Label: 13C(6)15N(4)]	1026.5454	513.7762 (2+)	
PTPδ meA1⁻A2⁺	L	SGGTPIR	687.3784	334.1928 (2+)	y^{3}, y^{5}, y^{6}
	H	SGGTPIR [Label: 13C(6)15N(4)]	697.3867	349.1970 (2+)	
PTPδ meA1⁺A2⁻	L	SESIGALQIEQSEESDQGK	2034.9462	1017.9767 (2+)	y^{10}, y^{11}, y^{12}
	H	SESIGALQIEQSEESDQGK [Label: 13C(6)15N(2)]	2042.9604	1021.9838 (2+)	
PTPδ exon 60	L	NVLELNDVR	1071.5793	536.2936 (2+)	y^{5}, y^{6}, y^{7}
	H	NVLELNDVR [Label: 13C(6)15N(4)]	1081.5876	541.2977 (2+)	
PTPδ exon 61	L	VVAVNNIGR	941.5527	471.2803 (2+)	y^{5}, y^{6}, y^{7}
	H	VVAVNNIGR [Label: 13C(6)15N(4)]	951.5610	476.2844 (2+)	

Supplementary Table 5. Summary of the LAR-RPTP microexon profiles identified in the current study

Region	LAR-RPTP microexons					
	Ptprs meA	*Ptprs* meB	*Ptprd* meA	*Ptprd* meB	*Ptprf* meA	*Ptprf* meB
**Cortex**	98.9	75.0	54.5	3.3	50.7	85.2
**Olfactory bulb**	92.5	79.1	54.5	3.6	59.3	74.0
**Thalamus**	98.2	53.4	45.2	14.5	47.2	95.9
**Hippocampus**	98.4	77.3	42.1	13.4	72.5	79.5
**Striatum**	97.8	62.5	47.5	18.2	58.1	89.3
**Cerebellum**	98.7	66.5	60.9	15.3	68.3	90.7

Region	Cell type	LAR-RPTP microexons					
		Ptprs meA	*Ptprs* meB	*Ptprd* meA	*Ptprd* meB	*Ptprf* meA	*Ptprf* meB
**Cortex**	Excitatory neuron	95.7	42.8	49.9	2.0	34.5	91.9
	SST+ interneuron	89.9	92.1	17.1	31.6	0.6	92.6
	PV+ interneuron	80.0	5.6	13.1	18.9	1.0	91.9
**Olfactory bulb**	Excitatory neuron	94.1	88.4	43.9	14.2	46.5	92.3
	SST+ interneuron	96.6	4.9	48.2	27.3	23.0	2.2
	PV+ interneuron	92.7	7.8	71.9	34.7	90.2	1.4
**Thalamus**	Excitatory neuron	90.8	0.2	63.5	17.6	74.1	98.8
	SST+ interneuron	99.2	11.1	44.0	11.1	98.4	95.0
	PV+ interneuron	98.2	50.9	9.2	17.6	99.8	0.4
**Hippocampus**	Excitatory neuron	95.9	28.0	39.9	9.9	34.5	7.2

	SST+ interneuron	90.7	12.7	22.8	11.4	59.1	0.4
	PV+ interneuron	87.4	13.5	36.03	11.6	29.3	91.1
**Striatum**	SST+ interneuron	75.5	29.0	16.1	25.1	98.9	38.6
	PV+ interneuron	88.2	93.8	29.8	15.8	86.5	5.5
	Drd1+ dopaminergic neuron	89.3	30.2	45.9	17.4	97.2	92.9
	Drd2+ dopaminergic neuron	97.5	22.5	2.1	42.8	97.8	39.7

**Projection from the indicated input regions**		**LAR-RPTP microexons**					
**Target region**	**Input neuron region**	*Ptprs* meA	*Ptprs* meB	*Ptprd* meA	*Ptprd* meB	*Ptprf* meA	*Ptprf* meB
Medial prefrontal cortex	Cortex	1.1	33.3	10.6	3.7	89.7	85.1
	Thalamus	92.8	86.3	13.3	34.9	78.8	92.2
	Hippocampus	0.4	82.1	36.7	3.8	90.7	92.0
	Striatum	1.0	24.7	38.0	0.8	92.3	89.7
Hippocampal CA1	CA3	98.0	1.0	39.5	32.3	99.0	2.5
	Subiculum	99.4	92.9	44.2	16.4	98.9	2.3
	Entorhinal cortex	99.0	2.4	53.3	45.2	98.9	76.9

Supplementary references

1. Treutlein, B., Gokce, O., Quake, S.R. & Südhof, T.C. Cartography of neurexin alternative splicing mapped by single-molecule long-read mRNA sequencing. *Proc Natl Acad Sci U S A* **111**, E1291-1299 (2014).
2. Schreiner, D., *et al.* Targeted combinatorial alternative splicing generates brain region-specific repertoires of neurexins. *Neuron* **84**, 386-398 (2014).
3. Fuccillo, M.V., *et al.* Single-Cell mRNA Profiling Reveals Cell-Type-Specific Expression of Neurexin Isoforms. *Neuron* **87**, 326-340 (2015).
4. Földy, C., *et al.* Single-cell RNAseq reveals cell adhesion molecule profiles in electrophysiologically defined neurons. *Proc Natl Acad Sci U S A* **113**, E5222-5231 (2016).
5. Wang, X., *et al.* Three-dimensional intact-tissue sequencing of single-cell transcriptional states. *Science* **361**, eaat5691 (2018).
6. Furlanis, E., Traunmuller, L., Fucile, G. & Scheiffele, P. Landscape of ribosome-engaged transcript isoforms reveals extensive neuronal-cell-class-specific alternative splicing programs. *Nat Neurosci* **22**, 1709-1717 (2019).
7. Lukacsovich, D., *et al.* Single-Cell RNA-Seq Reveals Developmental Origins and Ontogenetic Stability of Neurexin Alternative Splicing Profiles. *Cell Rep* **27**, 3752-3759 (2019).
8. Attilio, P.J., *et al.* Transcriptomic Analysis of Mouse Brain After Traumatic Brain Injury Reveals That the Angiotensin Receptor Blocker Candesartan Acts Through Novel Pathways. *Front Neurosci* **15**, 636259 (2021).
9. Yao, Z., *et al.* A transcriptomic and epigenomic cell atlas of the mouse primary motor cortex. *Nature* **598**, 103-110 (2021).
10. Parada, G.E., *et al.* MicroExonator enables systematic discovery and quantification of microexons across mouse embryonic development. *Genome Biol* **22**, 43 (2021).

Uncropped scans of all DNA-PAGE and immunoblot gel images

Fig. 1 Profiling of LAR-RPTP microexon expression repertoires by targeted deep RNA sequencing

Figure 1f

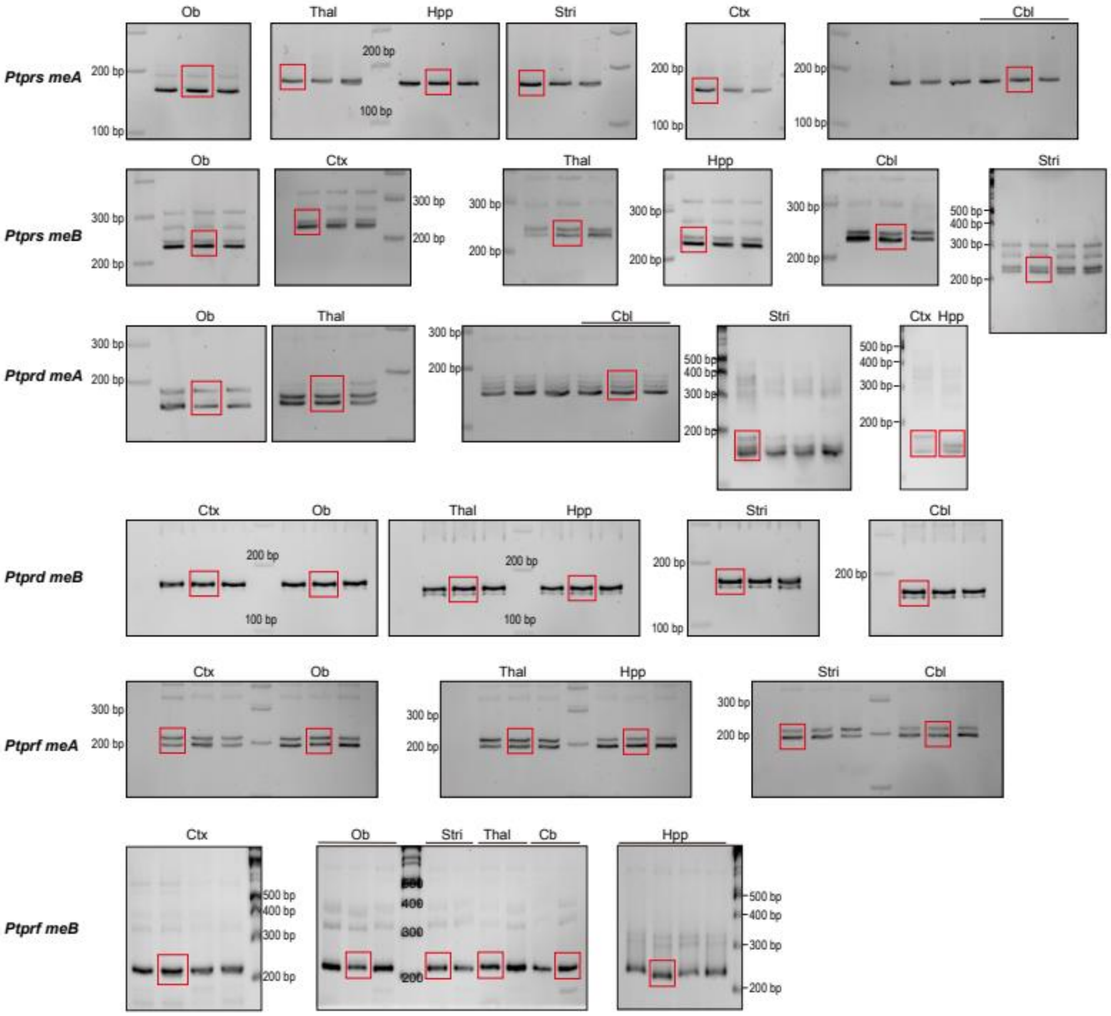

Fig. 2 Profiling the PTPδ meA+ proteoform by targeted proteomics

Figure 2e

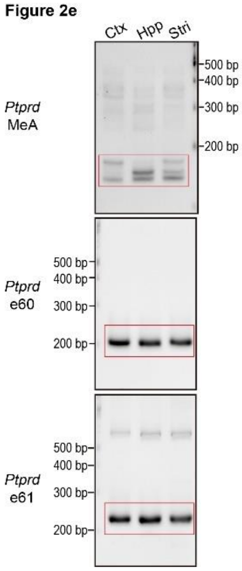

Fig. 3 Profiling of cell-type–specific LAR-RPTP microexon expression repertoires

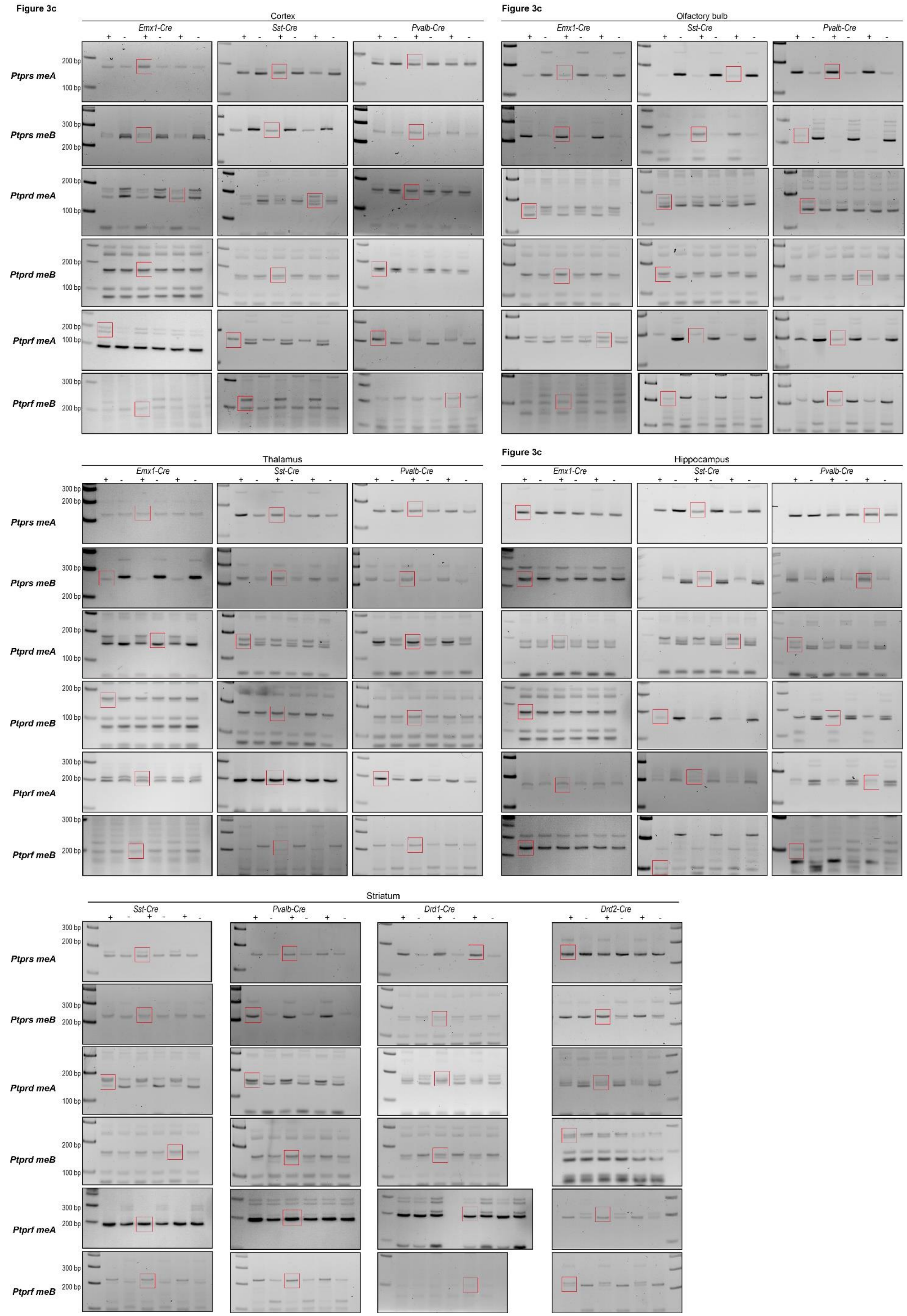

Fig. 4 Profiling of circuit-type–specific LAR-RPTP microexon expression repertoires

Figure 4e

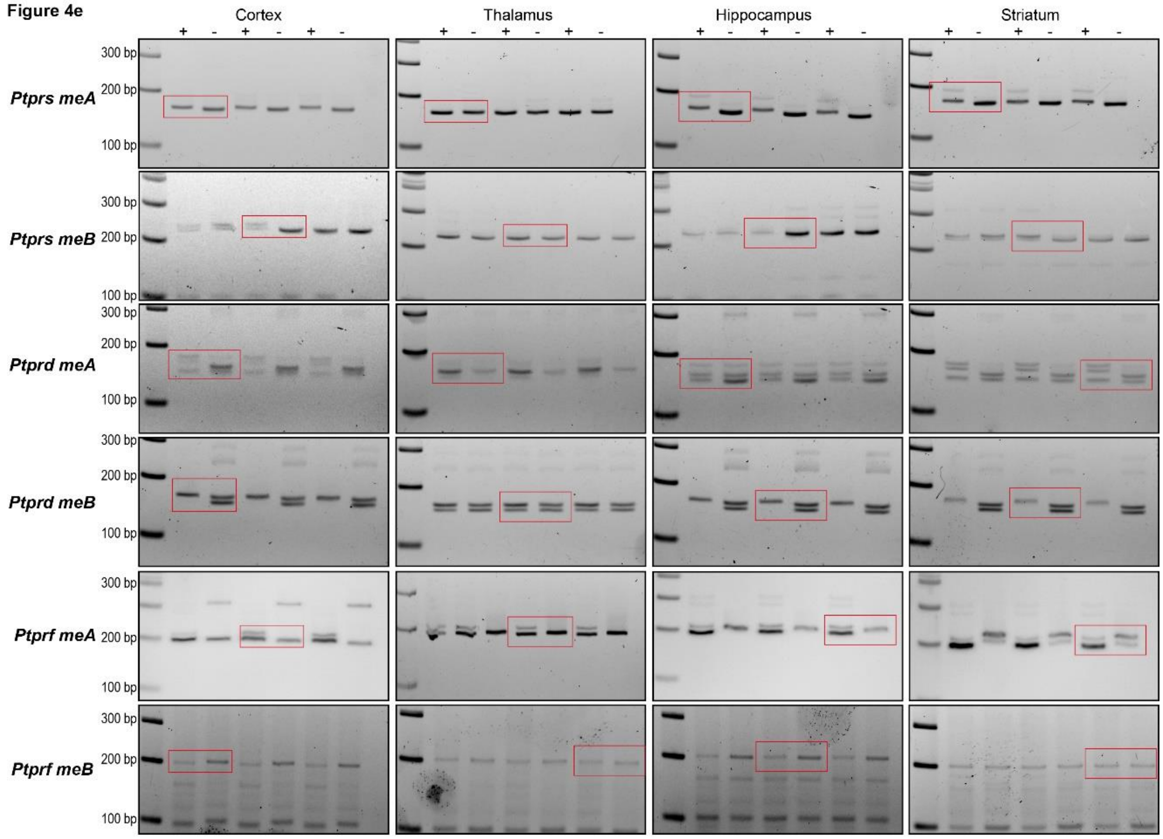

Figure 4f

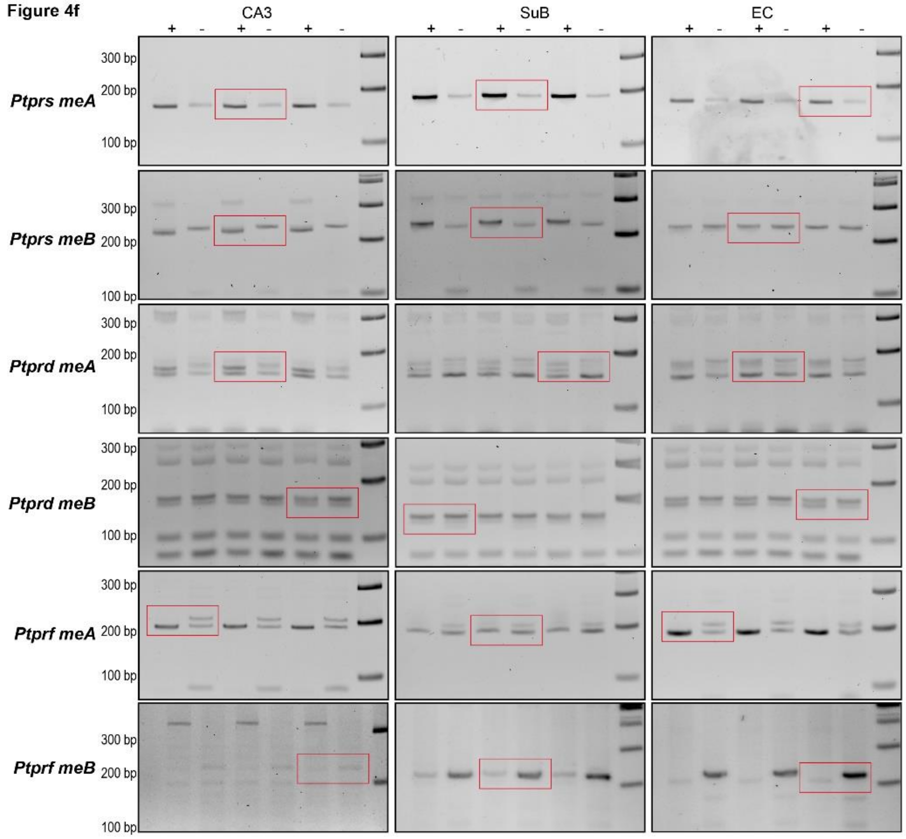

Fig. 5 Increased *Ptprd* meA microexon inclusion in fear memory engrams of the adult mouse DG

Figure 5c

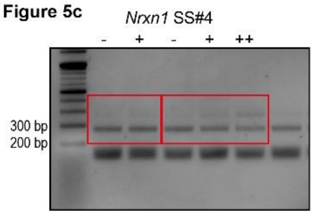

Figure 5e

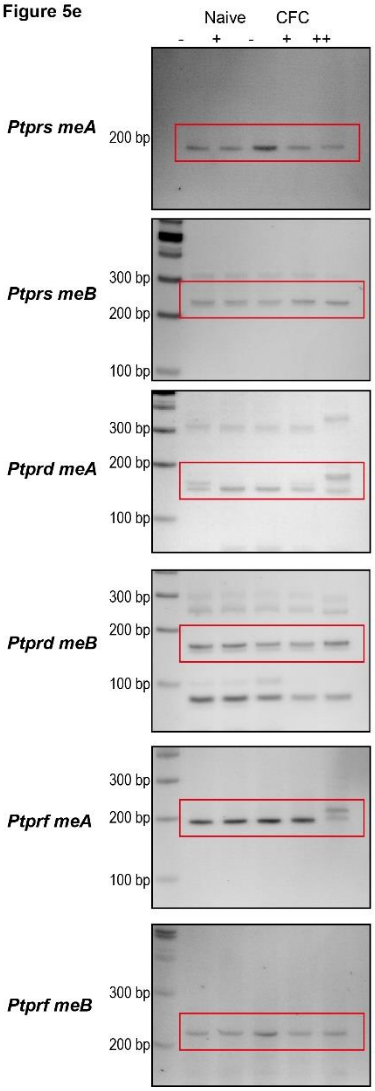

Fig. 6 Effects of neural circuit-specific deletion of PTPδ on the specific glutamatergic synaptic properties of hippocampal CA1 pyramidal neurons

Figure 6b

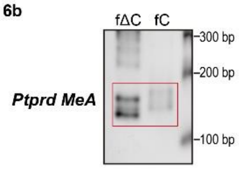

Fig. 7 Presynaptic PTPδ employs meA Microexon inclusion for differential regulation of distinct excitatory synaptic transmission in hippocampal CA1 neural circuits

Figure 7b

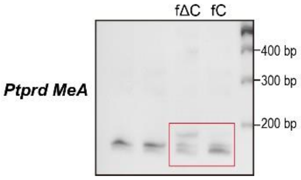

Fig. 9 Activity-triggered SuB-CA1 circuit-specific changes of PTPδ meA insertion modulates NMDA receptor-mediated responses and object-location memory in mice

Figure 9d

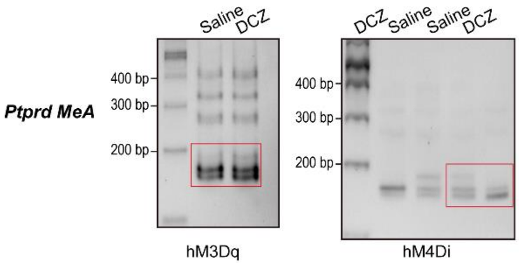

Supplementary Figure 1. Determination of the cutoff for MAJIQ-based alternative splicing analysis, analyses of brain region-specific Nrxn1 splicing at SS#4, and alternative splicing landscapes of LAR-RPTP mRNAs

Supplementary Figure 1

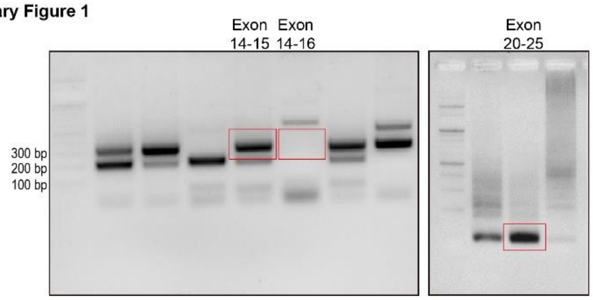

Supplementary Figure 3. Expression profiles of mouse LAR-RPTP microexons in male and female brains

Supplementary Figure 3a

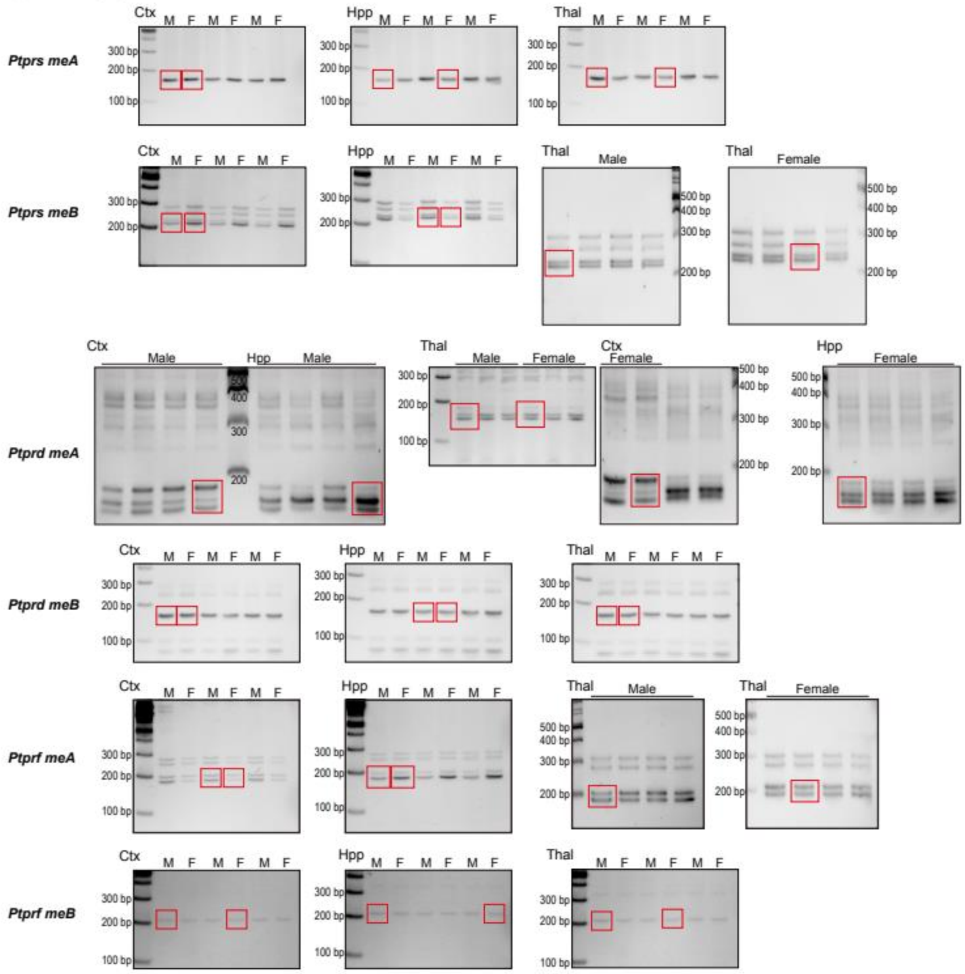

Supplementary Figure 4. Authenticity testing of anti-PTPδ and anti-PTPσ antibodies, LC-MS/MS spectra of various PTPδ-derived peptides expressed in the cortex of adult mice by Shotgun mass spectrometry analysis, and spectra of PTPδ meA1+ meA2+ peptides expressed in the cortex, hippocampus and striatum of adult mice by PRM analysis

Supplementary Figure 4b

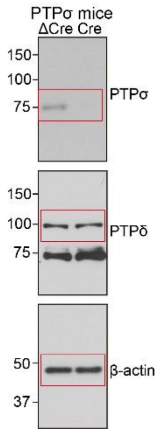

Supplementary Figure 4c

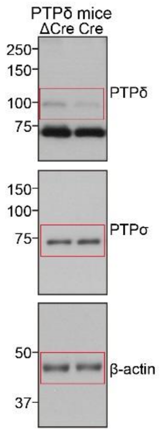

Supplementary Figure 5. Profiling of mouse LAR-RPTP microexon profiles in distinct hippocampal CA1 GABAergic interneurons

Supplementary Figure 5c

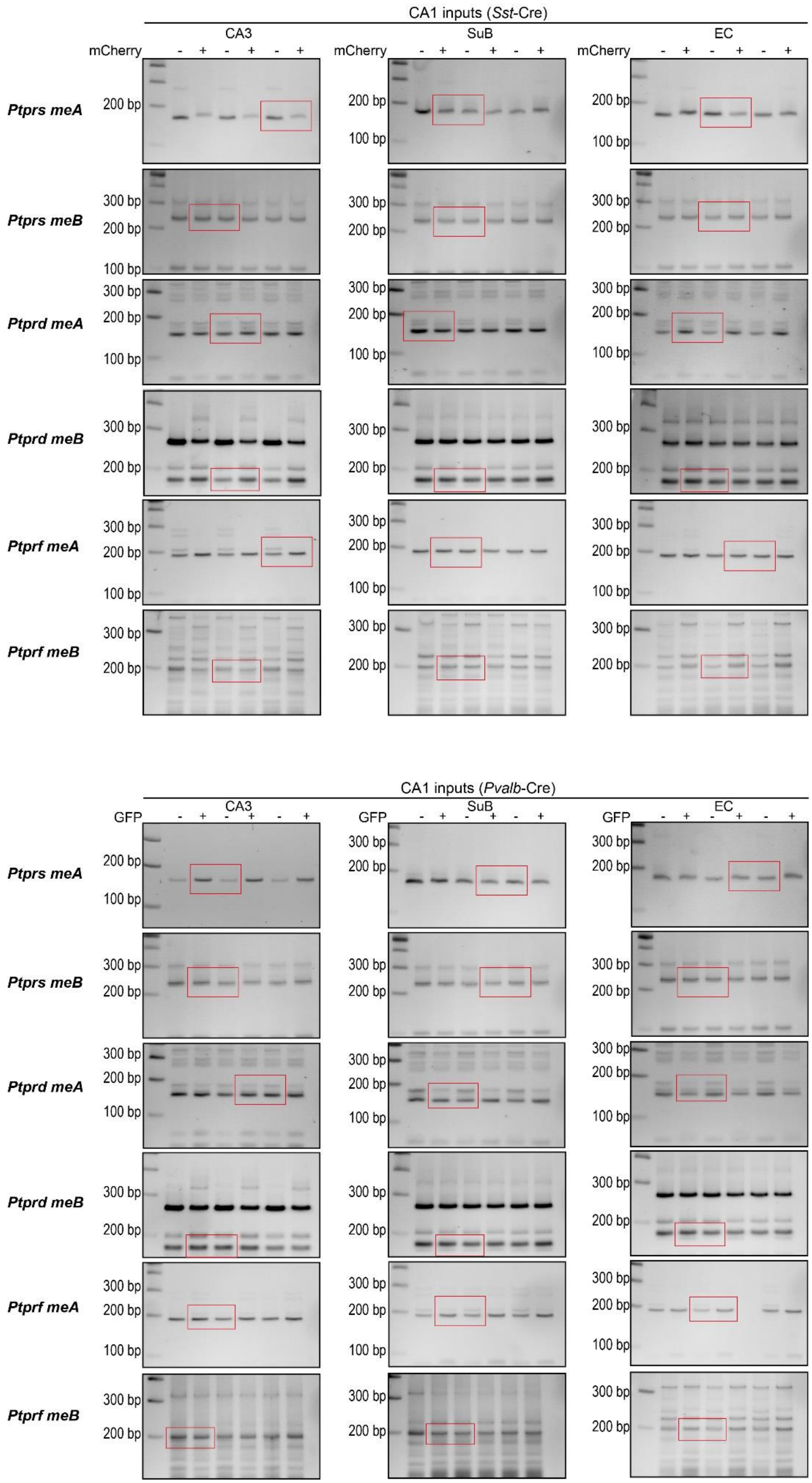

Supplementary Figure 6. Validation of the loss of Ptprd variants containing meA in cortical cells of PTPδ meA floxed mice expressing Cre recombinase, expression of PTPδ protein in three brain regions that innervate hippocampal CA1 neurons, and distribution of retrogradely labeled input neurons in the CA3, SuB or EC that project to dCA1 neurons

Supplementary Figure 6a

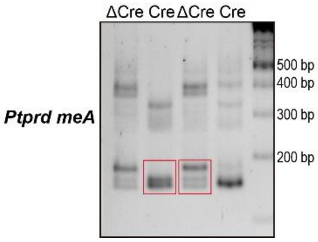

Supplement: Supplementary file 1 — Supplementary Information [file 41467_2024_45695_MOESM1_ESM.pdf]
