# Supplementary material for: Specification of neural circuit architecture shaped by context-dependent patterned LAR-RPTP microexons
Source: Nat Commun. 2024 Feb 22;15:1624. doi: 10.1038/s41467-024-45695-0 (PMC10883964; doi:10.1038/s41467-024-45695-0)
Supplement: Supplementary file 3 — Inventory of Supporting Information [file 41467_2024_45695_MOESM3_ESM.docx]

**Inventory of Supporting Information**

**9 Supplementary Figures**

**Supplementary Figure 1.** Determination of the cutoff for MAJIQ-based alternative splicing analysis, analyses of brain region-specific *Nrxn1* splicing at SS#4, and alternative splicing landscapes of LAR-RPTP mRNAs

**Supplementary Figure 2.** Isoform abundance of LAR-RPTP mRNAs, as assessed using kallisto

**Supplementary Figure 3.** Expression profiles of mouse LAR-RPTP microexons in male and female brains

**Supplementary Figure 4.** Authenticity testing of anti-PTPσ and anti-PTPδ antibodies, LC-MS/MS spectra of various PTPδ-derived peptides expressed in the cortex of adult mice by Shotgun mass spectrometry analysis, and spectra of PTPδ meA1^+^ meA2^+^ peptides expressed in the cortex, hippocampus and striatum of adult mice by PRM analysis

**Supplementary Figure 5.** Profiling of mouse LAR-RPTP microexon profiles in distinct hippocampal CA1 GABAergic interneurons

**Supplementary Figure 6.** Validation of the loss of *Ptprd* variants containing meA in cortical cells of PTPδ meA floxed mice expressing Cre recombinase, expression of PTPδ protein in three brain regions that innervate hippocampal CA1 neurons, and distribution of retrogradely labeled input neurons in the CA3, SuB or EC that project to dCA1 neurons

**Supplementary Figure 7.** Analysis of PTPδ function in regulating specific GABAergic neural circuits in the mouse hippocampal CA1

**Supplementary Figure 8.** Behavioral analyses of hippocampal circuit-specific *Ptprd*-cKO mice

**Supplementary Figure 9.** Viral tracing reveals that presynaptic elimination of PTPδ meA^+^ variants in the SuB🡪dCA1 circuit does not affect the projection of SuB neurons onto dCA1 neurons

**5 Supplementary Tables**

**Supplementary Table 1**. Prior studies investigating microexons of *neurexin* and LAR-RPTP mRNAs

**Supplementary Table 2.** Cross-correlation score (X_corr_) of chemically synthesized peptides used in this study

**Supplementary Table 3.** Oligonucleotide sequences for genotyping and RT-PCR experiments described in this study

**Supplementary Table 4**. Information on peptides used for LC-PRM analyses

**Supplementary Table 5.** Summary of the LAR-RPTP microexon profiles identified in the current study

**Source Data files**

**1. Uncropped scans of all DNA-PAGE and immunoblot gel images**

**2. Data values presented in quantitative presentations of all figures**

**3. Reporting Summary**

**4. Editorial Policy Checklist**
