# Supplementary material for: Specification of neural circuit architecture shaped by context-dependent patterned LAR-RPTP microexons
Source: Nat Commun. 2024 Feb 22;15:1624. doi: 10.1038/s41467-024-45695-0 (PMC10883964; doi:10.1038/s41467-024-45695-0)
Supplement: Supplementary file 4 — Reporting Summary [file 41467_2024_45695_MOESM4_ESM.pdf]

Corresponding author(s): JAEWON KO
Last updated by author(s): Dec 26, 2023

Reporting Summary

Nature Portfolio wishes to improve the reproducibility of the work that we publish. This form provides structure for consistency and transparency in reporting. For further information on Nature Portfolio policies, see our Editorial Policies and the Editorial Policy Checklist.

Statistics

For all statistical analyses, confirm that the following items are present in the figure legend, table legend, main text, or Methods section.

n/a	Confirmed	
☐	☒	The exact sample size (n) for each experimental group/condition, given as a discrete number and unit of measurement
☐	☒	A statement on whether measurements were taken from distinct samples or whether the same sample was measured repeatedly
☐	☒	The statistical test(s) used AND whether they are one- or two-sided<br>*Only common tests should be described solely by name; describe more complex techniques in the Methods section.*
☒	☐	A description of all covariates tested
☐	☒	A description of any assumptions or corrections, such as tests of normality and adjustment for multiple comparisons
☒	☐	A full description of the statistical parameters including central tendency (e.g. means) or other basic estimates (e.g. regression coefficient) AND variation (e.g. standard deviation) or associated estimates of uncertainty (e.g. confidence intervals)
☐	☒	For null hypothesis testing, the test statistic (e.g. F, t, r) with confidence intervals, effect sizes, degrees of freedom and P value noted<br>*Give P values as exact values whenever suitable.*
☒	☐	For Bayesian analysis, information on the choice of priors and Markov chain Monte Carlo settings
☒	☐	For hierarchical and complex designs, identification of the appropriate level for tests and full reporting of outcomes
☒	☐	Estimates of effect sizes (e.g. Cohen's d, Pearson's r), indicating how they were calculated

Our web collection on statistics for biologists contains articles on many of the points above.

Software and code

Policy information about availability of computer code

Data collection	Data were collected using the following softwares: Zen v2.3 (Zeiss), Clampex 10/Multiclamp 700B (Molecular Devices), EthoVision XT10.5 (Noldus), Illumina HiSeq 2500 (Illumina), IP2 search algorithm (Integrated Proteomics Applications, Inc., San Diego) and FACS Aria III (BD).
Data analysis	Data were analyzed using the following softwares: Excel (Microsoft), Prism v8 (GraphPad), Image Lab (BIO-RAD), Image J (NIH), STAR (version 2.6.0a) (Dobin et al., 2013), MAJIQ (MAJIQ Vaquero-Garcia et al., 2016), Kallisto (Bray et al., 2016), pCLAMP version 11.0 (Molecular Devices), OriginPro 8.5 software (OriginLab), DTASelect software (Tabb et al., 2002) (The Scripps Research Institute, San Diego, CA, USA), Skyline daily 22.2.1.425 (MacCross Lab Software), and FlowJo (BD research Cloud).

For manuscripts utilizing custom algorithms or software that are central to the research but not yet described in published literature, software must be made available to editors and reviewers. We strongly encourage code deposition in a community repository (e.g. GitHub). See the Nature Portfolio guidelines for submitting code & software for further information.

Data

Policy information about availability of data

All manuscripts must include a data availability statement. This statement should provide the following information, where applicable:

- Accession codes, unique identifiers, or web links for publicly available datasets
- A description of any restrictions on data availability
- For clinical datasets or third party data, please ensure that the statement adheres to our policy

The raw RNA-seq data generated in this study have been deposited in the NCBI BioProject under accession code PRJNA943606 (https://dataview.ncbi.nlm.nih.gov/object/PRJNA943606?reviewer=qb6tg4pvrhm5gr7rk468b5l63l). The proteome data generated in this study have been deposited in the MassIVE database under accession code MSV000091879 (https://massive.ucsd.edu/ProteoSAFe/dataset.jsp?task=1e036c7781474927b9e5593d32feb7f9). A reporting summary for this article is available as a Supplementary Information file. The main data supporting the findings of this study are available within the article and its Supplementary Figures. The source data underlying Figs. 1–9, Supplementary Fig. 1–3, Supplementary Fig. 5 and Supplementary Figs. 7–9 is provided as a Source Data file. Additional details on datasets and protocols that support the findings of this study will be made available by the corresponding author upon reasonable request. Source data are provided with this paper. The data sets presented in this study are included in full wherever possible, and source data are provided within this paper.

Research involving human participants, their data, or biological material

Policy information about studies with human participants or human data. See also policy information about sex, gender (identity/presentation), and sexual orientation and race, ethnicity and racism.

Reporting on sex and gender	n/a
Reporting on race, ethnicity, or other socially relevant groupings	n/a
Population characteristics	n/a
Recruitment	n/a
Ethics oversight	n/a

Note that full information on the approval of the study protocol must also be provided in the manuscript.

Field-specific reporting

Please select the one below that is the best fit for your research. If you are not sure, read the appropriate sections before making your selection.

☒ Life sciences ☐ Behavioural & social sciences ☐ Ecological, evolutionary & environmental sciences

For a reference copy of the document with all sections, see nature.com/documents/nr-reporting-summary-flat.pdf

Life sciences study design

All studies must disclose on these points even when the disclosure is negative.

Sample size	Sample sizes are indicated in the figure legends and were determined based on historical practices in our lab, as previously reported (Han et al., iScience 2020; Kim et al., Cell Rep 2021; Kim et al., PNAS 2022). Careful effort was made whenever possible to perform averaging across true biological replicates rather than pseudo-replicates. No power analysis or other statistical method was used to predetermine sample sizes.
Data exclusions	No data were excluded from the analyses.
Replication	At least three independent biological repeats were conducted for all experiments. Data representative of the results are shown in the figures.
Randomization	Allocation was random in most experiments. Mice of the experimental and control groups were randomly counterbalanced.
Blinding	All experimenters collecting data were blind to genetic manipulations or treatment. All analyses were performed in a blinded manner. Mouse cohorts were grouped randomly at the time of weaning, as described in the Methods.

Reporting for specific materials, systems and methods

We require information from authors about some types of materials, experimental systems and methods used in many studies. Here, indicate whether each material, system or method listed is relevant to your study. If you are not sure if a list item applies to your research, read the appropriate section before selecting a response.

Materials & experimental systems

n/a	Involved in the study
☐	☒ Antibodies
☐	☒ Eukaryotic cell lines
☒	☐ Palaeontology and archaeology
☐	☒ Animals and other organisms
☒	☐ Clinical data
☒	☐ Dual use research of concern
☒	☐ Plants

Methods

n/a	Involved in the study
☒	☐ ChIP-seq
☐	☒ Flow cytometry
☒	☐ MRI-based neuroimaging

Antibodies

Antibodies used

The following antibodies were used at the indicated concentrations:
1. Rabbit polyclonal anti-PTPδ (customized antibody; JK123; 1μg/ml)
2. Rabbit polyclonal anti-PTPσ (customized antibody; JK125; 1μg/ml)
3. Goat polyclonal anti-EGFP (Rockland; Cat# 600-101-215; 1:1000)
4. FITC-AffiniPure donkey anti-goat IgG antibodies (Jackson ImmunoResearch; Cat# 705-095-147; RRID: AB_2340401; 1:150)

Validation

1 and 2. The JK123 and JK125 antibodies were validated by immunoblotting of mouse lysates from each KO mouse line (presented in Supplementary Figure 4b of the current paper).
3. Goat polyclonal anti-EGFP (Rockland; Cat# 600-101-215) was vendor-validated for immunohistochemistry using E5.5 Hex-GFP transgenic mouse embryo. and has been referenced by several papers (relevant information is indicated in the vendor's website).
4. FITC-AffiniPure donkey anti-goat IgG antibodies (Jackson ImmunoResearch; Cat# 705-095-147) were extensively validated by vendor and previous papers from our lab.

Eukaryotic cell lines

Policy information about cell lines and Sex and Gender in Research

Cell line source(s)

HEK293T cells were purchased from ATCC (Cat# CRL-3216).

Authentication

The cell line was not authenticated (other than by morphology and passage).

Mycoplasma contamination

After receiving a cell line into the laboratory and/or after > 10 passages, mycoplasma testing was performed using a Universal Mycoplasma Detection Kit (purchased from ATCC). The utilized cell lines tested negative for mycoplasma contamination.

Commonly misidentified lines
(See ICLAC register)

No commonly misidentified lines were used.

Animals and other research organisms

Policy information about studies involving animals; ARRIVE guidelines recommended for reporting animal research, and Sex and Gender in Research

Laboratory animals

The following mouse strains were used:
PTPσ conditional knockout (cKO) (Han et al., 2020)
PTPδ cKO (Park et al., 2020)
PTPδ meA cKO (Park et al., 2020)
PTPδ-tdTomato reporter (Park et al., 2020)
Rosa26LSL-tdTomato (Ai9; Cat# 007909; Jax)
Sst-IRES-Cre (Cat# 013044; Jax)
Emx1-Cre (Cat# 005628; Jax)
Pvalb-Cre (Cat# 008069; Jax)
Drd1-Cre (Cat# 3836633; Jax)
Drd2-Cre (Cat# 3836635; Jax)
Pvalb-T2A-FlpO-D (Cat #022730; Jax)
Sst-IRES-FlpO (Cat# 028579; Jax)
Wild-type C57BL6/N mice (Daehan Biolink)

All mice were maintained and handled in accordance with the animal care standards outlined in the Guide for the Care and Use of Experimental Animals and were approved by the Daegu Gyeongbuk Institute of Science and Technology (DGIST) Administrative Panel on Laboratory Animal Care (DGIST-IACUC-20102205-0003 and DGIST-IACUC-21060201-0010). Male adult mice on a C57BL/6N background were used for all studies (except those presented in supplementary Fig. 3). Mice were maintained at 24°C on a 12:12-h

	light/dark cycle, with lights on at 7:00 and off at 19:00. Mice were given ad libitum access to food and water. Mice were weaned on postnatal day 28 (P28), and 2–5 mice were housed per cage to avoid social isolation and overcrowding. Wild-type C57BL/6N background mice were purchased from Daehan Biolink.
Wild animals	This study did not involve wild animals.
Reporting on sex	All experiments were performed using male mice, except those presented in Supplementary Figure 3. Data were not disaggregated by sex.
Field-collected samples	This study did not involve field-collected samples.
Ethics oversight	All procedures were conducted in accordance with the animal care standards outlined in the guide for the care and use of experimental animals and were approved by the Daegu Gyeongbuk Institute of Science and Technology (DGIST) Administrative Panel on Laboratory Animal Care (DGIST-IACUC-19052109-00).

Note that full information on the approval of the study protocol must also be provided in the manuscript.

Flow Cytometry

Plots

Confirm that:

☒ The axis labels state the marker and fluorochrome used (e.g. CD4-FITC).

☒ The axis scales are clearly visible. Include numbers along axes only for bottom left plot of group (a 'group' is an analysis of identical markers).

☒ All plots are contour plots with outliers or pseudocolor plots.

☒ A numerical value for number of cells or percentage (with statistics) is provided.

Methodology

Sample preparation	Mouse brains were dissociated using a papain dissociation system (Worthington; Cat# LK003150). Dissected brain tissues were minced and incubated in SEBSS containing papain (20 units/μL), DNase (2000 units/μL), and actinomycin-D (5 μg/ml; Sigma; Cat# 50-76-0) at 37°C for 1.5 hours with gentle shaking. Debris and broken cells were removed by centrifugation, and cell pellets were suspended in cold SEBSS containing actinomycin-D and subjected to FACS.
Instrument	FACS Aria III (BD)
Software	FACS Aria III (BD), FlowJo
Cell population abundance	Distinct fluorescence-positive neuronal populations ranging from 10^5 to 10^9 cells were sorted until 200,000-300,000 events were acquired. Considering the variation in cell types used in this study, approximately 1-10% of a given sample population was subjected to analysis.
Gating strategy	Fluorescence-negative control littermate brain tissues were dissociated in parallel and used as negative controls. The specific neural population was obtained by FACS; fluorescence intensity was used to select the targeted neural population, and cell size gating was based on a FSC (forward scatter) level set to exclude cell doublets and non-cell particles.

☐ Tick this box to confirm that a figure exemplifying the gating strategy is provided in the Supplementary Information.
